# Presence of TMPRSS2-ERG is associated with alterations of the metabolic profile in human prostate cancer

**DOI:** 10.18632/oncotarget.9817

**Published:** 2016-06-03

**Authors:** Ailin Falkmo Hansen, Elise Sandsmark, Morten Beck Rye, Alan J. Wright, Helena Bertilsson, Elin Richardsen, Trond Viset, Anna M. Bofin, Anders Angelsen, Kirsten M. Selnæs, Tone Frost Bathen, May-Britt Tessem

**Affiliations:** ^1^ Department of Circulation and Medical Imaging, Faculty of Medicine, NTNU, Norwegian University of Science and Technology, Trondheim, Norway; ^2^ Department of Cancer Research and Molecular Medicine, Faculty of Medicine, NTNU, Norwegian University of Science and Technology, Trondheim, Norway; ^3^ St. Olavs Hospital, Trondheim, Norway; ^4^ Department of Urology, St. Olavs Hospital, Trondheim, Norway; ^5^ Cancer Research UK Cambridge Institute, University of Cambridge, Cambridge, United Kingdom; ^6^ Department of Medical Biology, UiT - The Arctic University of Norway, Tromsø, Norway; ^7^ Department of Pathology and Medical Genetics, St. Olavs Hospital, Trondheim, Norway; ^8^ Department of Laboratory Medicine, Children's and Women's Health, Faculty of Medicine, NTNU, Norwegian University of Science and Technology, Trondheim, Norway

**Keywords:** metabolomics, citrate, spermine, HR-MAS, MRSI

## Abstract

TMPRSS2-ERG has been proposed to be a prognostic marker for prostate cancer. The aim of this study was to identify changes in metabolism, genes and biochemical recurrence related to TMPRSS2-ERG by using an integrated approach, combining metabolomics, transcriptomics, histopathology and clinical data in a cohort of 129 human prostate samples (41 patients). Metabolic analyses revealed lower concentrations of citrate and spermine comparing ERG_high_ to ERG_low_ samples, suggesting an increased cancer aggressiveness of ERG_high_ compared to ERG_low_. These results could be validated in a separate cohort, consisting of 40 samples (40 patients), and magnetic resonance spectroscopy imaging (MRSI) indicated an *in vivo* translational potential. Alterations of gene expression levels associated with key enzymes in the metabolism of citrate and polyamines were in consistence with the metabolic results. Furthermore, the metabolic alterations between ERG_high_ and ERG_low_ were more pronounced in low Gleason samples than in high Gleason samples, suggesting it as a potential tool for risk stratification. However, no significant difference in biochemical recurrence was detected, although a trend towards significance was detected for low Gleason samples. Using an integrated approach, this study suggests TMPRSS2-ERG as a potential risk stratification tool for inclusion of active surveillance patients.

## INTRODUCTION

The genetic fusion between the erythroblast transformation-specific (*ETS*) transcriptional factor ETS-related gene (*ERG*) and the androgen-responsive promotor transmembrane protease, serine 2 (*TMPRSS2*) [1] is suggested to be a major mechanism driving prostate carcinogenesis. The TMPRSS2-ERG gene fusion is the most common gene rearrangement in prostate cancer [2], with a reported prevalence of 15-78% [3]. Presence of the gene fusion is the main reason for overexpression of *ERG* which is further associated with epithelial-to-mesenchymal potential, cell invasion and cell proliferation [4].

From the initial discovery in 2005 [5], the TMPRSS2- ERG gene fusion has been linked to clinical outcome parameters such as early onset of prostate cancer [6], negative outcome in watchful waiting patients [7–9] and a higher risk of disease progression in active surveillance patients [10]. However, considering the prognostic value of TMPRSS2-ERG in prostatectomy patients, most studies find no association to outcome after surgery [6, 11–13]. In a meta-analysis of 5,074 prostatectomy specimens, there were no associations between the presence of TMPRSS2-ERG and biochemical recurrence or lethal disease [14]. Although the clinical significance of TMPRSS2-ERG is yet to be proven, presence of the fusion gene is a key genomic event specific for prostate cancer that may be of importance for risk assessment or treatment stratification of prostate cancer patients.

Metabolic markers may be indicative of aggressive disease and provide diagnostic and therapeutic information for improved characterization and stratification of prostate cancer patients. Lower levels of citrate and spermine have previously been linked to higher Gleason grade and more aggressive prostate cancer [15]. Citrate and spermine, including choline and creatine are metabolites detectable by *in vivo* patient magnetic resonance spectroscopy imaging (MRSI), which imply a potential for transferring biomarkers to a clinical setting [16]. A recent study revealed ERG-specific metabolic alterations, particularly connected to fatty acid oxidation [17] and an earlier study found increased glucose uptake to be related to the metabolic sensor neuropeptide gamma (*NPY*) in ERG rearrangement positive prostate cancer [18]. Apart from these two studies, the relationship between cancer metabolism and TMPRSS2-ERG remains unexplored.

The integration of transcriptomic data with metabolomics and histopathology is a promising tool for gaining important molecular information, in order to understand states and pathways of disease. In this study, we used prostatectomy tissue samples obtained through a standardized harvesting protocol [19] where metabolic and gene expression data are collected after histopathology evaluation [20] in order to integrate data from transcriptomics, metabolomics and histopathology. Prostate tissue samples were analyzed by HR-MAS (high resolution magic angle spinning) MRS (magnetic resonance spectroscopy), followed by detection of the fusion gene using gene expression microarray measurements for the main cohort, and fluorescence *in situ* hybridization (FISH) for an independent validation cohort. HR-MAS is a non-destructive method, which permits gene expression analysis and histology to be performed on the exact same tissue sample, providing an excellent basis for correlating metabolic findings with concordant alterations in the transcriptome. The main objective of this study was to combine these techniques to investigate presence of the TMPRSS2-ERG gene fusion in two cohorts of human prostate cancer tissue and to identify its association to metabolism and biochemical recurrence.

## RESULTS AND DISCUSSION

The presence of TMPRSS2-ERG or expressing high ERG levels was in our prostate cancer patient cohorts associated with metabolic alterations and concordant changes of gene expression levels related to key metabolic genes. In two independent patient cohorts, we observed a decrease in concentrations of citrate and spermine in fusion positive and ERG_high_ patients, indicating increased aggressiveness according to previous findings on prostate cancer metabolism [15]. In addition, this relationship was significant within low Gleason samples which propose an early patient stratification possibility based on the fusion status and metabolic biomarkers.

### Presence of TMPRSS2-ERG/high ERG status

A 2 mm transversal prostate tissue slice was collected from 41 patients and from each slice several samples (median: 3, range: 1 to 6 per slice, depending on tumor size) were collected from cancerous and adjacent benign areas, in total 95 cancer and 34 benign samples, and termed the main cohort. Among the cancer samples, 34 of 95 (35.8%) were classified as ERG_high_, and were expected to possess the TMPRSS2-ERG fusion gene, while 30 (31.6%) and 31 (32.6%) were classified as ERG_low_ and ERG_intermediate_, respectively. In addition, 34 (26.4%) of the 129 samples in the cohort were classified as benign samples. The proportions harboring the fusion gene are in the lower range of the reported prevalence of 15-78% [3].

Generally, samples obtained from the same prostate, were all placed in the same ERG group or the adjacent ERG group. However, out of the 41 patients, 6 (14.6%) patients had samples belonging to all three ERG groups (Supplementary Table S1), which is in consistence with previously reports of ERG interfocal heterogeneity [21, 22]. Three patients had no cancer samples, leaving 38 patients as the main focus of this study. In order to validate our results, a second cohort of 90 prostate cancer patients was included, consisting of one needle biopsy sample per patient obtained after radical prostatectomy. Only 40 of the needle biopsies contained cancer and were included in the present study. In the validation cohort, 7 out of 40 patients, (17.5%) were fusion positive, while 33 out of 40 (82.5%) were fusion negative. The lower prevalence of TMPRSS2-ERG in the validation cohort may be due to a lower amount of tumor in the samples (median cancer content 40% and 70% in the validation and main cohort, respectively) and sampling only one sample per patient may fail to detect presence of TMPRSS2-ERG present in other parts of the prostate. Sample characteristics of both cohorts are presented in Table 1.

**Table 1:** Clinical characteristics for samples in the main- and validation cohort

	Main cohort			Validation cohort	
	ERG_low_	ERG_intermediate_	ERG_high_	TMPRSS2-ERG negative	TMPRSS2-ERG positive
**Prevalence**	30	31	34	33	7
**Gleason score of tissue samples**					
0	0	0	0	0	0
6	7	8	9	5	1
3 + 4	5	8	8	11	3
4 + 3	4	7	9	9	0
8	8	2	5	3	2
9	6	6	3	2	1
10	0	0	0	2	0
Not evaluated	-	-	-	1	-
**Cancer content (%) mean (range)**	59 (10 to 90)	66 (20 to 90)	64 (20 to 85)	38 (5 to 80)	36 (5 to 80)
**Stroma content (%) mean (range)**	28 (5 to 50)	23 (0 to 70)	26 (10 to 50)	39 (20 to 70)	45 (10 to 65)
**Benign epithelial content (%) mean (range)**	13 (0 to 50)	11 (0 to 30)	10 (0 to 40)	26 (0 to 40)	26 (10 to 30)
**Luminal space (%) mean (range)**	9 (0 to 32)	6 (0 to 30)	8 (0 to 21)	4 (0 to 14)	5 (0 to 13)

### Metabolic alterations associated with TMPRSS2-ERG/high ERG status

**Figure 1:**
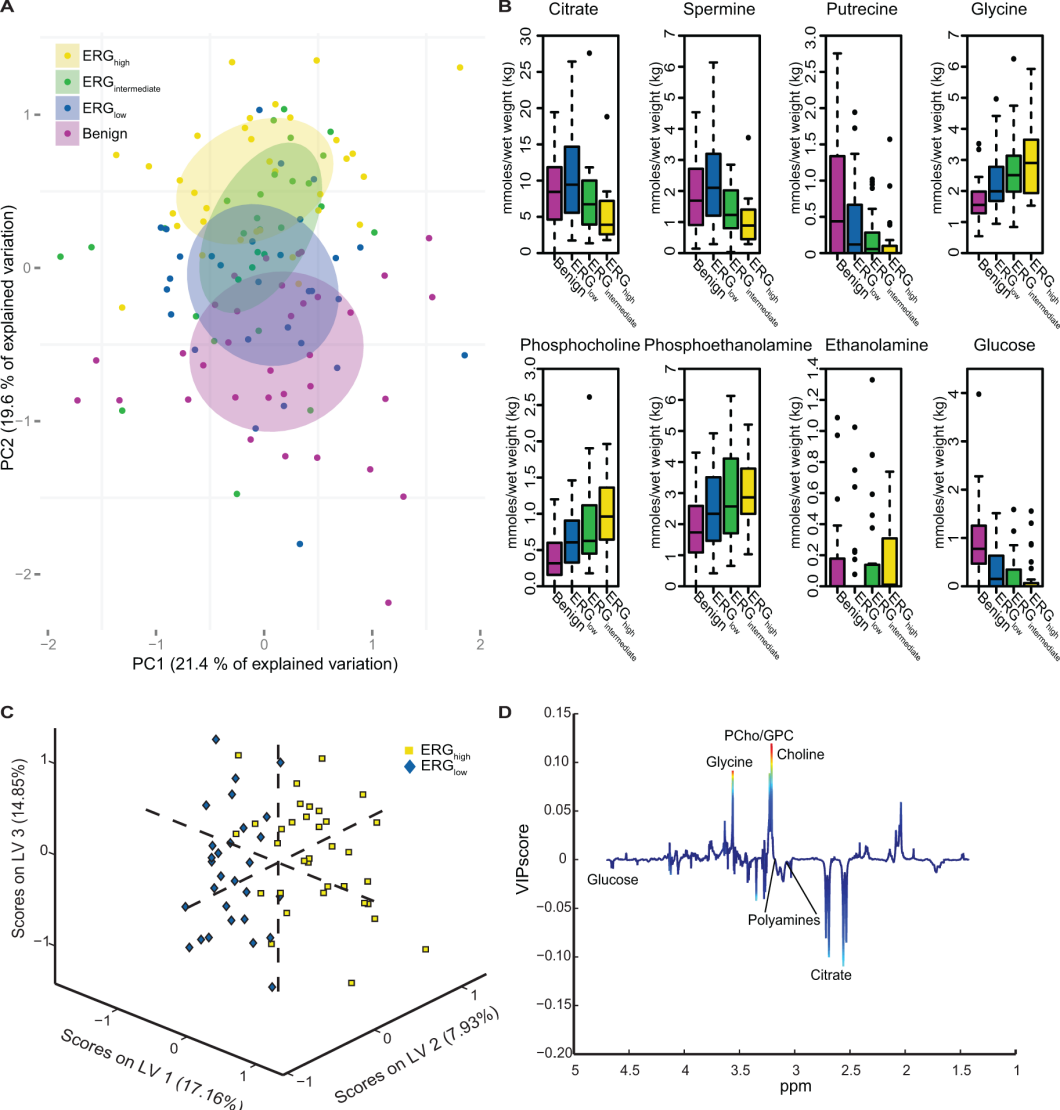
Multivariate analysis of spectral data and absolute quantification reveals metabolic differences between ERG groups (**A**) Principal component analysis (PCA) reveals a trend in the distribution of the metabolic clusters of metabolic profile from benign samples (purple) across ERGlow (blue) and ERGintermediate (green) to ERGhigh (yellow). (**B**) Absolute quantification of 23 metabolites showed significant trend across cancer samples, from ERGlow, (blue) through ERGintermediate (green) to ERGhigh (yellow) for eight of the metabolites. Increasing trends were found for glycine, phosphocholine, phosphoethanolamine, and ethanolamine. Decreasing trends were found for citrate, spermine, putrescine and glucose. Benign (purple) samples are shown for comparisons. (**C**) A partial least squares discriminant analysis (PLS-DA) model was able to separate ERGhigh (yellow) and ERGlow (blue) with a accuracy of 77 %, *p* < 0.001. (**D**) Loadings plot of latent variable 1 (LV1) indicate lower levels of citrate and the polyamines and higher levels of choline-containing metabolites comparing ERGhigh to ERGlow. The loadings are colored according to the variable importance in the projection (VIP) scores.

Unsupervised multivariate analysis of the metabolic profiles of the main cohort revealed a trend of clustering with respect to the three different ERG groups and the benign samples (Figure 1A). Significant trends across increasing ERG groups (cancer samples) were detected for the levels of citrate, spermine, putrescine, ethanolamine, glucose, glycine, phosphocholine and phosphoethanolamine (Figure 1B and Table 2). In normal prostate cells citrate is accumulated, while in prostate cancer, citrate is decreased or depleted [23]. Additionally, the normal prostate cells have one of the highest concentrations of polyamines in the body [24], and the polyamines are important for a variety of functions within the cell such as e.g. apoptosis, cell proliferation and differentiation [25, 26]. Decreasing levels of citrate, spermine and putrescine with increasing ERG status, suggested increased aggressiveness [15] of higher ERG status groups compared to lower ERG status groups. The increased levels of ethanolamine, phosphocholine and phosphoethanolamine further suggest an increased aggressiveness of the higher ERG status, as increased concentrations of choline-associated metabolites have been reported in prostate cancer, and are important in proliferation as structural components of cellular membranes [27, 28]. Glycine may also be important considering previous findings in breast cancer, suggesting it to be a marker of lower survival rates [29].

**Table 2:** Differences in levels of quantified metabolites in the main cohort comparing ERG_high_ samples with ERG_low_ samples

Metabolites	ERG_low_ *n* = 30	ERG_intermediate_ *n* = 31	ERG_high_ *n* = 34	ERG_high_vs ERG_low_ (*p*-values)	ERG_high_vs ERG_low_ (adjusted *p*-values)	*p*-trend
	*Concentrations mmoles/kg wet weight, median (IQR)*	*Concentrations mmoles/kg wet weight, median (IQR)*	*Concentrations mmoles/kg wet weight, median (IQR)*			
Citrate	9.44 (5.56 to 14.68)	6.74 (3.94 to 10.34)	3.91 (2.59 to 7.20)	< 0.001	< 0.001	< 0.001
Ethm	0 (0 to 0)	0 (0 to 0.15)	0.01 (0 to 0.31)	0.490	0.663	0.043
Glucose	0.15 (0.00 to 0.63)	0.00 (0.00 to 0.43)	0.00 (0.00 to 0.07)	0.008	0.061	0.007
Glycine	1.99 (1.68 to 2.78)	2.51 (1.98 to 3.18)	2.90 (1.93 to 3.65)	0.023	0.115	0.008
PCh	0.61 (0.33 to 0.91)	0.63 (0.43 to 1.17)	0.96 (0.64 to 1.36)	0.067	0.248	0.005
PE	2.33 (1.46 to 3.51)	2.57 (1.67 to 4.14)	2.86 (2.33 to 3.79)	0.087	0.248	0.032
Putrescine	0.12 (0 to 0.67)	0.06 (0 to 0.30)	0 (0 to 0.10)	0.025	0.115	0.003
Spermine	2.10 (1.20 to 3.19)	1.23 (0.79 to 2.02)	0.89 (0.45 to 1.40)	< 0.001	< 0.001	< 0.001

Ethm: Ethanolamine, PCh: Phosphocholine, PE: Phosphoethanolamine, IQR: Interquartile range. Adjusted *p*-values are adjusted using Benjamini-Hochberg correction for multiple testing.

Comparable results were found building a partial least squares discriminant analysis (PLS-DA) model based on the metabolic profiles where ERG_high_ was separated from ERG_low_ with an accuracy of 77% (sensitivity: 79%, specificity: 74%), *p* < 0.001 (Figure 1C). This proves that the metabolic profiles of samples which are expected to possess the fusion gene are well separated from those most likely not to harbor the gene rearrangement. Further, the loading plot for the latent variable 1 (LV1) (Figure 1D), explaining which metabolites that are important for the separation along LV1, showed decreased levels of citrate and polyamine levels in ERG_high_ compared to ERG_low_, while levels of choline-containing compounds were higher, supporting the hypothesis of a more aggressive phenotype of fusion positive prostate cancers.

**Figure 2:**
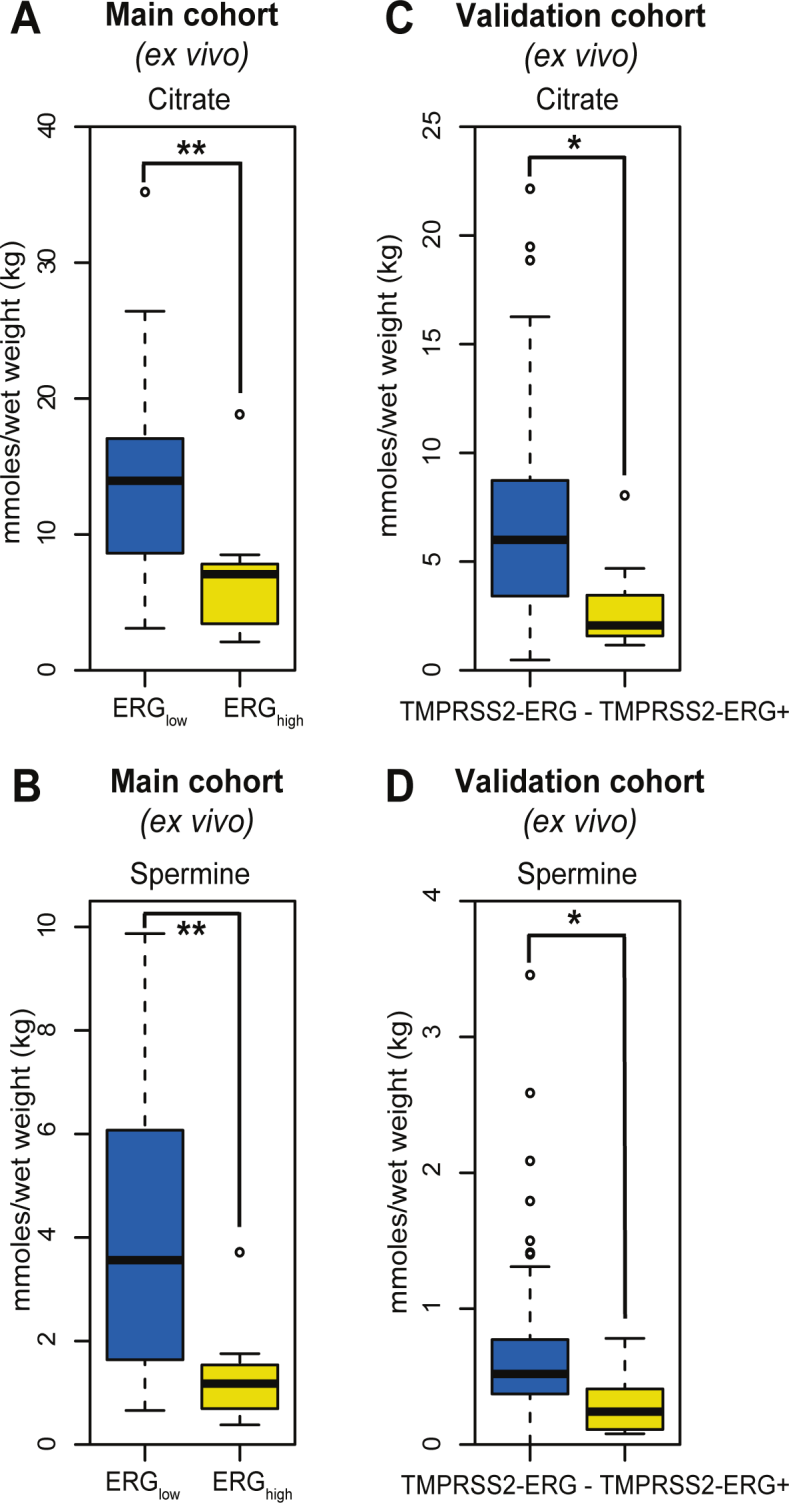
Box-plots for citrate and spermine comparing ERGhigh and ERGlow samples in the main cohort (*ex vivo*) and fusion positive and fusion negative patients in the validation cohort (**A**) Decreased levels of citrate were found comparing ERGhigh to ERGlow samples in the main cohort, *p* < 0.001, (**B**) Decreased levels of spermine were found comparing ERGhigh to ERGlow samples in the main cohort, *p* < 0.001, (**C**) Decreased levels of citrate were found comparing fusion positive to fusion negative patients in the validation cohort, *p* = 0.013, (**D**) Decreased levels of spermine were found comparing fusion positive to fusion negative patients in the validation cohort, *p* = 0.021.

Among the 23 quantified metabolites in the main cohort, the concentrations of citrate, spermine, putrescine and glucose were significantly decreased in ERG_high_ samples compared to ERG_low_, while the concentrations of glycine were significantly increased (Supplementary Table S2). However, after multiple testing corrections, only citrate and spermine were significant (Figure 2A and 2B, Table 2). In addition, our study revealed similar metabolic levels between ERG_low_ and benign samples (Supplementary Table S3), possibly confounded by effects of tissue heterogeneity [30], especially differences in stromal content between cancer and benign samples. Similar metabolic levels of citrate and spermine have previously been found comparing low Gleason grade and benign samples [15]. Despite the low prevalence of the fusion gene in the validation cohort, significantly decreased concentrations of citrate and spermine were detected in fusion positive samples. However, these differences were not significant after corrections for multiple testing, possibly due to the relatively small patient cohort, variations in the amount of cancer tissue between samples and the low number of fusion positive samples in this cohort (Figure 2C and 2D, Supplementary Table S4). A recent study [17] supports our metabolic findings by presenting significantly increased levels of glycerophosphoethanolamine, glycine, isoleucine, leucine and glutamate between ERG positive and ERG negative patients, and significantly decreased levels of myo-inositol, creatine, citrate, glucose, spermine and putrescine. However, we were not able to reveal any metabolic changes related to glycerophosphoethanolamine, isoleucine, leucine, glutamate and myo-inositol suggested by Meller et al. [17].

### Targeted analyses of key metabolic pathways

Due to the observed citrate and spermine changes, we performed targeted analyses of genes related to the polyamine pathway and citrate. We also investigated metabolic pathways connected to glycine and glucose metabolism, as TMPRSS2-ERG has been suggested to be linked to increased glucose uptake [17, 18].

### The polyamine pathway

**Figure 3:**
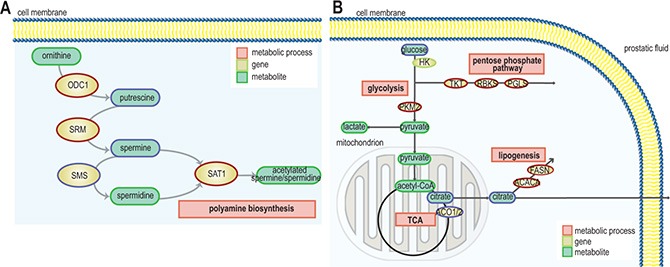
Schematic representation of pathways and gene expression levels of associated key enzymes altered due to presence of the fusion gene (**A**) the polyamine pathway, gene/protein names: ODC1: ornithine decarboxylase 1, SRM: spermidine synthase, SMS: spermine synthase, SAT1: spermidine/spermine N1-acetyltransferase 1 (blue = down-regulation, red = up-regulation) and (**B**) TCA cycle, fatty acid synthesis and pentose phosphate pathway. ACO1/2: aconitase 1/2, ACACA: acetyl-CoA carboxylase alpha, FASN: fatty acid synthase, HK: hexokinase, PKM2: pyruvate kinase, PGLS: 6-phosphogluconolactonase, RBKS: ribokinase, and TKT: transketolase (blue = down-regulation, red = up-regulation).

Expression of polyamine pathway genes were found to be increased in ERG_high_ samples compared to ERG_low_, where spermidine synthase (*SRM*) and spermidine N(1)-acetyltransferase (*SAT1*) displayed the highest significance (Figure 3A and Supplementary Table S5). Especially, the strong upregulation of *SAT1* leads to a rapid depletion of cellular spermidine and spermine [31], which is in agreement with the low concentrations of spermine observed in ERG_high_ compared to ERG_low_. In addition, overexpression of *SRM* without concordant upregulation of ornithine decarboxylase (*ODC1*) will lead to reduced levels of putrescine which was observed in the present study. *ODC1* overexpression is reported frequently among prostate cancer patients [32, 33], where it mediates the conversion of ornithine to putrescine which is the rate-limiting enzyme of the polyamine pathway. However, this does not seem to be the main mode of regulation in ERG_high_ versus ERG_low_ samples in our cohort, where changes in *SAT1* and *SRM* seem to be the main drivers of altered polyamine metabolism.

### Citrate and fatty acid synthesis

In the present study, we found that a significantly decreased expression of *ACO2* in the TCA cycle (Figure 3B and Supplementary Table S5) is linked to a phenotype characterized by low levels of citrate in ERG_high_ tissue samples. Franklin and Costello [34] suggested that normal prostate epithelial cells are citrate-producing, but become citrate-oxidizing following transformation to malignant cells, and that *ACO2* is the key enzyme for this transformation. Decreased expression of *ACO2* have been linked to increased citrate secretion [35], causing higher levels of citrate which can be redirected to the cytosol, contributing to restore acetyl-CoA and oxaloacetate pools. Our results indicate that citrate is shunted out to the cytosol where it may be used for *de novo* synthesis of fatty acids to meet the high demands for building blocks for biosynthesis in cancer, as we observed an increased expression of the key lipogenic enzymes acetyl-CoA carboxylase alpha (*ACACA*) and fatty acid synthase (*FASN*) in ERG_high_ tissue samples. Additionally, a higher expression of long-chain acyl-CoA synthetase3 (*ASCL3*) was detected, which is previously suggested to cause lipid accumulation [36]. High expression of *FASN*, have been found increased in several types of cancers, including prostate cancer and is strongly correlated with malignant transformation and poor prognosis [37, 38]. Increased fatty acid synthesis is suggested to be a key feature of prostate cancer suggesting aggressiveness of disease [38], and the increased lipogenic profile of ERG_high_ samples supports the hypothesis of an increased aggressiveness with presence of TMPRSS2-ERG.

### Glucose, glycine and pentose phosphate pathway

A significant reduction of glucose was found prior to correction for multiple testing, comparing ERG_high_ with ERG_low_. We detected a differential expression of NPY, in line with results from a previous study [18], comparing ERG_high_ and ERG_low_ samples, where lower levels of glucose were connected to a phenotype with a higher expression of *NPY*. Our results indicate that ERG_high_ samples have lower glucose levels or are rapidly consuming glucose and thus lowering the detectable glucose levels. Moreover, there was not an increased concentration of lactate in ERG_high_ compared to ERG_low_, and both increased and decreased expression of key enzymes within glycolysis and the TCA were detected (Figure 3B and Supplementary Table S5). However, a highly significant increased expression of oxoglutarate dehydrogenase-like (*OGDHL*), a key control point in the TCA, was found in ERG_high_ compared to ERG_low_. Interestingly, the increased expression of pyruvate kinase (*PKM2*) may slow glycolysis and redirect carbohydrate intermediates to e.g. the pentose phosphate pathway (PPP). This is supported by overexpression of key enzymes both in the oxidative and the reductive part of the PPP, specifically the expression of 6-phosphogluconolactonase (*PGLS*), transketolase (*TKT*), and ribokinase (*RBKS*) (Supplementary Table S5). Collectively, these results suggest that glucose may be shunted into the PPP among ERG_high_ samples. The PPP provides nucleotide precursors and helps regenerate NADPH which is important for maintaining the redox state and for supporting the synthesis of fatty acids for cancer cells [39].

When investigating the most central genes associated with the metabolism of glycine we did not find any possible explanation for the increased levels of glycine among ERG_high_ compared to ERG_low_ samples (Supplementary Table S5).

### Risk stratification based on the presence of TMPRSS2-ERG

Risk stratification for choice of treatment in low grade prostate cancer is currently a challenge. We therefore investigated the possibility to stratify patients according to the presence of fusion/ERG status within low Gleason samples (Gleason score ≤ 3 + 4). We detected pronounced differences both in metabolism and gene expression levels between ERG_high_ and ERG_low_, restricted to low Gleason samples (Supplementary Table S8). A significant decrease in the concentrations of citrate, spermine, putrescine and glycerophosphoethanolamine were detected in ERG_high_ samples compared to ERG_low_ samples, and a significant increase were found in glutamine and glycine after multiple testing corrections (Supplementary Table S8). However, restricting our analyses to high Gleason samples (Gleason score ≥ 4 + 3), only significantly decreased concentrations of citrate and spermine were observed comparing ERG_high_ and ERG_low_ (Supplementary Table S9). Expression levels of key enzymes in the metabolism of the polyamines, glucose and fatty acid displayed higher significance levels when restricting the analyses to low Gleason samples compared to high Gleason samples (Supplementary Table S6 and S7).

As both the metabolic and the gene expression levels were more pronounced in the low Gleason group, presence of the fusion gene may serve as a tool for risk- or treatment stratification of low Gleason patients. In high Gleason samples, we generally observed less significant metabolic and transcriptomic alterations due to ERG status. High Gleason score has been linked to genomic instability and multiple genetic alterations [40, 41]. As the high Gleason samples are heterogeneous, the transcriptomic- and metabolic differences between ERG_high_ and ERG_low_ may possibly be masked by the effect of other genetic alterations present among these samples.

**Figure 4:**
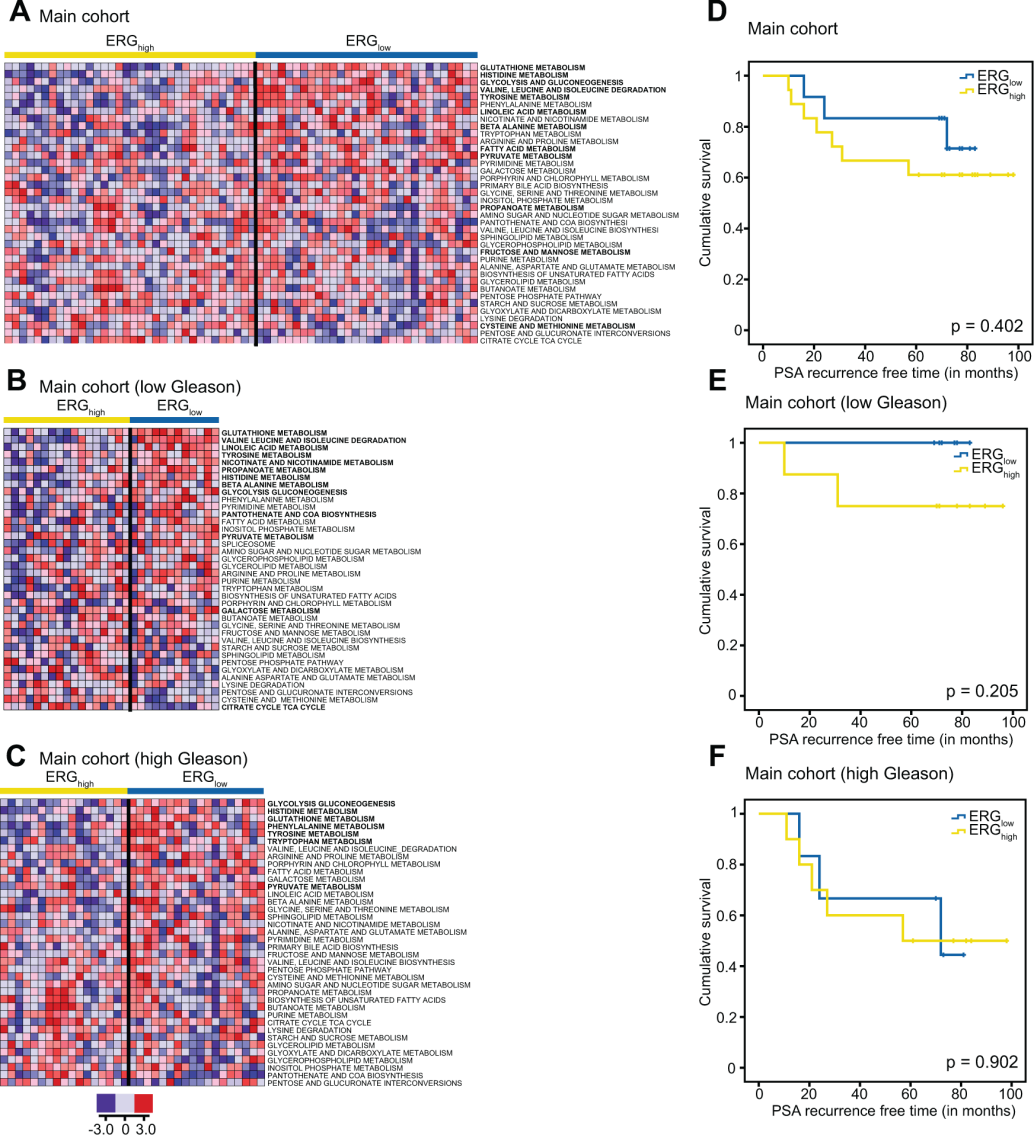
Single sample GSEA (ssGSEA) show alterations of several key metabolic pathways and biochemical recurrence for ERGhigh and ERGlow and Kaplan-Maier plots for biochemical recurrence ssGSEA results of key metabolic pathways for the main cohort (**A**) the low Gleason samples (**B**) and the low Gleason samples within the main cohort (**C**) where red indicates up-regulation of the given gene set and blue down-regulation of a given gene set. Pathways with significantly different (*p* < 0.05) ssGSEA-values comparing ERGhigh and ERGlow while adjusting for multiple samples per patient, are indicated in bold. Kaplan-Maier plots for ERGhigh and ERGlow in the main cohort (**D**), low Gleason patients (**E**) and high Gleason patients (**F**).

To increase the understanding of metabolism associated with the presence of the fusion gene, INMEX and ssGSEA analyses were performed, and indicated several metabolic pathway differences between ERG_high_ and ERG_low_ (Figure 4A) including glutathione (including polyamines), glycolysis, and additionally purine and pyrimidine, which are important precursors for nucleotides (Supplementary Table S10). In concordance with our findings in metabolic concentrations, both INMEX and GSEA showed more significant differences when the analyses were restricted to low Gleason samples (Figure 4B) than to high Gleason samples (Figure 4C). These results are presented in Supplementary Tables S11–S18.

In conclusion, metabolic alterations in the presence of the fusion gene are more pronounced in the low grade compared to aggressive cancer, and may be suggested as a possible risk stratification tool for low Gleason prostate cancer patients. Metabolism suggests a more aggressive phenotype connected to presence of the fusion gene. However, further studies on prognostics and validation are needed. Due to the small number of samples in the validation cohort, metabolic differences between low- and high Gleason samples could not be validated by this cohort.

### Biochemical recurrence and ERG status

Prognostics and biochemical recurrence connected to presence of the fusion gene have previously shown varying results [14]. At a median follow-up of 6.5 years in our study (range 1.8 to 8.3 years), 10 (33.3%) of the 30 patients with follow-up data had experienced biochemical recurrence (prostate-specific antigen (PSA) ≥ 0.2 ng/ml). No significant difference was observed in biochemical recurrence between ERG_high_ and ERG_low_ patients in the main cohort (Figure 4D–4F), which is in agreement with other studies on radical prostatectomy cohorts [6, 11–14]. However, there was a trend towards significance when restricting to the low Gleason patients, *p* = 0.205 (Figure 4E). Due to the low number of included patients, the current study may not have the sufficient statistical power to reveal significant differences in rate of biochemical recurrence between ERG_high_ and ERG_low_.

### Translational potential by *in vivo* patient magnetic resonance spectroscopy imaging

**Figure 5:**
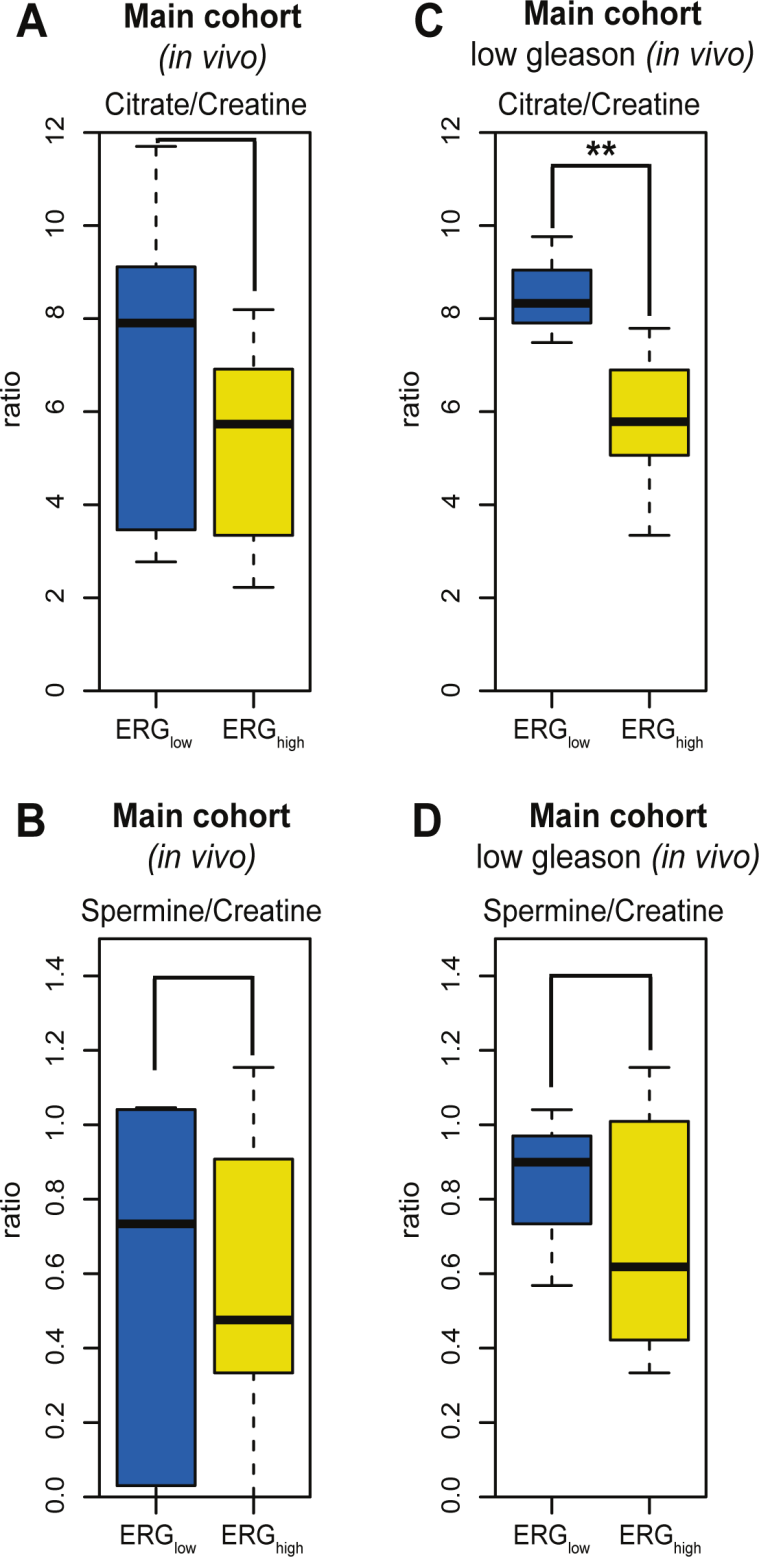
Box-plots for citrate and spermine comparing ERGhigh and ERGlow samples in the main cohort (*in vivo*) (**A**) No significant difference in levels of citrate/creatine in the main cohort (*in vivo* MRSI) between ERGhigh and ERGlow, *p* = 0.083, (**B**) Decreased levels of citrate/creatine were found comparing ERGhigh to ERGlow in the main cohort (*in vivo* MRSI), restricting to low Gleason samples, *p* < 0.001, (**C**) No significant difference in levels of spermine/creatine between ERGhigh and ERGlow in the main cohort (*in vivo* MRSI), *p*= 0.158, (**D**) No significant difference in levels of spermine/creatine were found comparing ERGhigh and ERGlow in the main cohort (*in vivo* MRSI), restricting to low Gleason samples, *p* = 0.094.

The potential of transferring biomarkers and knowledge to 3T and 7T *in vivo* patient MRSI [16], makes HR-MAS MRS on prostate tissue samples attractive for basic research. A subset of the patients in the main cohort (9 patients, 21 samples) had data from *in vivo* MRSI acquired prior to surgery. The *in vivo* spectroscopy voxels were spatially matched to the HRMAS tissue samples [16]. The *in vivo* citrate/creatine ratio from spatially matched voxels was decreased with borderline significance in ERG_high_ compared to ERG_low_, *p* = 0.083 (Figure 5A), while choline/creatine and spermine/creatine ratios were not significant, *p* = 0.667 and *p* = 0.158, respectively (Figure 5B). However, in the low Gleason group (5 patients, 11 samples), the citrate/creatine ratio was significantly decreased, *p* < 0.001 (Figure 5C) and in addition, the levels of choline/creatine ratio was significantly increased (*p* = 0.041), while the spermine/creatine ratio was borderline decreased (*p* = 0.094) in ERG_high_ compared to ERG_low_ (Figure 5D). Within high Gleason samples (4 patients, 10 samples), only a decreased choline/creatine ratio was significantly detected (*p* = 0.018), comparing ERG_high_ to ERG_low_. Alterations in MRSI *in vivo* measured citrate, choline and spermine levels may offer a possibility for stratification of low risk prostate cancer patients without the need of biopsies, and a possibility to enroll patients into active surveillance programs, with non-invasive MRSI monitoring.

### Concluding remarks

This study presents a distinct metabolic profile with concordant alterations of gene expression levels of key metabolic enzymes in prostate tissue samples with the presence of TMPRSS2-ERG. The metabolic profile was especially connected to the metabolism of polyamines and citrate, but also glycolysis and fatty acid metabolism. Our results indicate that TMPRSS2-ERG differentiates towards a phenotype that is associated with characteristics of an aggressive phenotype of prostate cancer. Additionally, the observed metabolic alterations can be translated to *in vivo* patient MRSI.

## MATERIALS AND METHODS

### Samples and patient cohorts

In the main cohort, a 2 mm transversal prostate tissue slice was collected from 48 prostate cancer patients after radical prostatectomy at St.Olavs Hospital, Trondheim, between 2007 and February 2010, with no previous treatment for prostate cancer, using a highly standardized harvesting method thoroughly described by Bertilsson et al. [15, 19]. From each tissue slice, several samples (average: 6, range: 4 to 11 per slice, depending on tumor size) were collected from cancerous and adjacent benign areas. In total 362 samples were extracted for RNA and acceptable RNA integrity number (RIN) scores were obtained from 354 samples. Samples with a high Gleason score, large extent of cancer and high quality RNA were prioritized. Seven patients were excluded either due to lack of cancer in the extracted samples (2 slices) or lack of quality of the samples in the microarray analyses (5 slices), and 4 samples were excluded due to low HR- MAS spectral quality. In total 95 cancer and 34 benign samples from 41 patients (median: 3, range: 1 to 6 per slice) were collected.

For validation of the results, a second cohort of 90 prostate cancer patients was included, consisting of one needle biopsy sample per patient obtained after radical prostatectomy. The samples were selected from a large biobank (~1000 patients, ~2000 samples) collected from prostate cancer patients after radical prostatectomy at St.Olavs Hospital, Trondheim, between 2007 and February 2010. The samples were selected from patients with highest tumor volume in order to collect tissue with high cancer content. The patients had not received any treatment for prostate cancer prior to sampling. Only 40 of the needle biopsies contained cancer and were included in the present study. The two cohorts were independent, i.e. no patient belonged to both cohorts. Sample characteristics for the main cohort and the validation cohort are given in Table 1. Both cohorts are approved by the Regional Committee of Medical and Health Research Ethics (REC), Central Norway, and all patients gave written, informed consent.

### HR-MAS MRS

^1^H HR-MAS MRS analysis was performed as previously described [15, 19]. Acquired spectral data were exponential Fourier transformed (line broadening 0.3 Hz), baseline- and phase-corrected using Topspin 3.2 (Bruker Biospin, Germany). Samples in the validation cohort were of equivalent weight (mean weight: 12.3 mg, range 6.7 to 21.9 mg) to samples in the main cohort (mean weight: 12.7 mg, range: 3.0 to 21.9 mg). Samples were hematoxylin- and eosin stained (HE, main cohort) or hematoxylin-eosin-saffron stained (HES, validation cohort) due to different routines in staining protocols at different times. HE/HES stained sections were used for histopathological evaluation of Gleason grading and assessment of cancer-, benign epithelial-, and stromal content. Two pathologists (TV and ER) evaluated the sections from the main cohort and an interrater agreement (κ) of 0.66, indicating substantial agreement, was found for distinguishing the samples into benign, low Gleason (Gleason score ≤ 3 + 4) and high Gleason (Gleason score ≥ 4 + 3). The first reading (TV) was used for grading in this study due to a slight degradation of the cryosections from the initial reading to the second reading. The validation cohort sections were evaluated by one pathologist (TV).

### Definition of ERG groups and combining transcriptomics and metabolomics data

Gene expression profiles from the main cohort were obtained as previously described by Bertilsson et al. [19, 20]. The microarray data has previously been published in Array Expression with access number: E-MTAB-1041. The gene expression data were log2 transformed and quantile normalized [20]. Gene Set Enrichment Analysis (GSEA) scores were calculated for detection of specific enrichment of the ERG-fusion gene set based on prostate cancer related gene sets [42] as previously described by Rye et al. [43]. GSEA focuses on gene sets, i.e. groups of genes that share common biological function, chromosomal location, or regulation and in order to detect pathway changes more sensitively [44]. Based on the overall ERG GSEA score the samples were classified as ERG_high_ if the score were increased two-fold compared to the mean ERG GSEA of the cancer samples. The rest of the cancer samples were equally divided into groups of ERG_low_ and ERG_intermediate._ The ERG_high_ samples were defined as possessing the highest probability for being fusion positive, while the ERG_low_samples were defined as having the lowest probability for being fusion positive. Due to uncertainties of the fusion status of the ERG_intermediate_ group, most of the differential analyses of metabolite and gene expression levels were performed comparing the ERG_high_ and ERG_low_ groups. Classification of samples per patient to the individual ERG groups is presented in Supplementary Table S1.

To link transcriptomic and metabolomics data connected to ERG status, we used two approaches; 1) integrative meta-analysis of expression data (INMEX) where lists of genes and metabolites (individually analyzed) are combined and significant genes and metabolites are mapped to Kyoto Encyclopedia of Genes and Genomes (KEGG) pathways [45], and where enrichment and topology analysis identify important pathways [46], 2) single sample GSEA (ssGSEA) [44] calculates separate enrichment scores for each pairing of a sample and gene set, which represents the degree of up- or down-regulation of a gene [47]. Enrichment of KEGG gene set collections in the Molecular Signatures Database (Broad Institute, version 5.0) were performed using the GSEA software (Broad Institute, version 2.0.14) [44, 48]. ssGSEA analyses were performed using ssGSEAprojection [47], and results from the 38 most relevant metabolic pathways were visualized using HeatMapViewer (version 13), using GenePattern (version 3.9.4) [49].

### Fluorescence *in situ* hybridization

The TMPRSS2-ERG status of samples in the validation cohort was determined by using a break-apart assay with a triple-labeled color commercial probe (Kreatech Diagnostics, The Netherlands). The probe detects the deletion between TMPRSS2 and ERG at 21q22. The FISH assay was carried out on 4 μm formalin-fixed, paraffin-embedded tissue sections after deparaffinization which were then pretreated using a commercial tissue section kit for paraffin-embedded tissue (Histology FISH Accessory Kit, Dako). The probe mix was applied and denatured at 80°C for 5 minutes before hybridization at 37°C overnight using a Dako hybridizer. The slides were counterstained with DAPI (4′,6-diamidino-2-phenylindole) from the Histology FISH Accessory kit. Results were visualized using a 100x oil immersion objective on a Nikon Eclipse 90i fluorescent microscope (Nikon Corp., Japan) equipped with appropriate filters. For each sample, 25 non-overlapping nuclei in cancer areas were evaluated for deletion of the TMPRSS2 (21q22) gene region associated with TMPRSS2-ERG. In order to compensate for nuclear truncation, the cut-off level for an informative result was defined as loss of the TMPRSS2 (21q22) gene region at least 80% of tumor cell nuclei.

### Luminal space measurements

Cryosections from the main cohort and the paraffin-embedded sections from the validation cohort were digitalized with 40x magnification and the luminal spaces were identified using a color-based segmentation (positive pixel count algorithm in ImageScope v8.0, Aperio Technologies) as described by Langer et al. [50].

### Quantification of metabolites

Individual metabolites in the HR-MAS spectra were quantified using LCModel [51] based on a basis set containing 23 metabolites generated using NMRSIM (Bruker BioSpin, Germany) as previously described by Giskeødegård et al. [15]. Similarly, a separate basis set of 25 metabolites was built for the validation cohort, adding glutathione and ascorbate to the basis set as improvements of the previous basis set. In both cohorts, metabolites were quantified according to known amounts of formate and reported as mmoles/kg wet weight.

### *In vivo* magnetic resonance spectroscopy imaging

As part of a previous published study [16], 9 patients (24 samples) in the main cohort had *in vivo* MRSI metabolic data from patients from spatially matched voxels to the tissue sampling sites. Due to the low number of samples, the samples were divided into two equal groups: ERG_high_ for samples with ERG score higher than the median of the cancer samples, and ERG_low_ for the samples with ERG score lower than median. Three samples were excluded due to low spectral quality of the associated MRSI spectrum. Details regarding e.g. acquisition and quantification of *in vivo* metabolite levels have previously been described in Selnæs et al. [16].

### Statistical analysis

The HR-MAS MRS spectra were baseline corrected and peak aligned using icoshift [52] in MATLAB r2013a (The Mathworks, Inc., USA). Contamination signals from ethanol (3.65-3.69 ppm) were removed before normalization by probabilistic quotient normalization (PQN) [53]. Principal component analysis (PCA) and partial least squares discriminant analysis (PLS-DA) were performed on the Carr-Purcell-Meiboom-Gill (CPMG) spectra between 1.46 and 4.66 ppm. Data were centered prior to analysis. To avoid overfitting, PLS-DA models were validated through a 5-fold random subset cross-validation, and repeated 10 times. The number of latent variables was chosen based on the first local minima of cross-validated classification error for PLS- DA. Permutation testing was performed to assess the significance of the multivariate models (*n* = 1000). PCA and PLS-DA models were built using mixOmics in R [54] and PLS_toolbox 7.8.2 (Eigenvector Research, Inc., USA) in MATLAB, respectively.

In the main cohort, comparisons of quantified metabolites and gene expression levels, including metabolite levels from *in vivo* MRSI were performed by using linear mixed models in Stata 13 (StataCorp, USA), accounting for the effect of several samples originating from the same patient. Gene expression levels for metabolic enzymes were mainly chosen according to their proximity and influence of the quantified metabolites found within KEGG pathways. For the polyamine pathway, genes previous reported to be central in polyamine metabolism, provided as the basis for the analyses [26]. A total of 63 genes were included in the study, and are listed as part of Supplementary Tables S5–S7. Comparisons of gene expression levels were performed using the most significant probe if several probes for the same gene were available. Adjusted linear mixed models were built by including the relative amount of stroma, benign epithelia, cancer tissue and luminal spaces as continuous covariates, in order to minimize the possible confounding effects of tissue heterogeneity. Adjusted models for gene expression data are presented in Supplementary Tables S5–S7, while adjusted models for metabolic data are presented in Supplementary Tables S2, S8 and S9. Test for trends of metabolite levels over ERG groups were performed using the nptrend function in Stata. In the validation cohort, comparisons of metabolite levels between TMPRSS2-ERG positive and negative samples were compared using Student *t*-test. *P*-values less than 0.05 were considered significant and *q*-values less than 0.05 were considered significant after corrections for multiple testing

Correlations between individual metabolites and tissue composition and relative luminal space were examined using Pearson's correlation, and correlations are presented in Supplementary Table S19. Corrections for multiple testing were performed by Benjamini-Hochberg correction. Multiple testing corrections were performed individually for the main- and the validation cohort, accounting for the number of comparisons for the metabolic data and the gene expression data individually. Prior to statistical analysis, all metabolite concentrations were transformed in order to obtain normal-distributed data or residuals. Type of transformation performed was based on visual inspections of resulting QQ-plots and histograms of the transformed data. Metabolic data were in general square-root transformed, except lactate which was log-transformed and glycine which was transformed by 1 divided by the square-root.

Differences in rates of biochemical recurrence (PSA ≥ 0.2 ng/ml) after prostatectomy were estimated with the Kaplan-Meier method and compared using the log rank statistics and the Cox proportional hazards regression model. Patients were classified as ERG_high_ if they had one or more samples within ERG_high_. Patients were followed from date of surgery until last measured PSA or death. Time to event was calculated as the time in months between date of surgery and date of PSA-blood collection indicating biochemical recurrence or date of last follow-up blood collection. In total, 30 patients classified as ERG_high_ or ERG_low_ were included in the analysis, while patients with only benign or ERG_intermediate_ samples were excluded from the analysis.

## SUPPLEMENTARY TABLES


