# Supplementary material for: Presence of TMPRSS2-ERG is associated with alterations of the metabolic profile in human prostate cancer
Source: Oncotarget. 2016 Jun 3;7(27):42071–85. doi: 10.18632/oncotarget.9817 (PMC5173117; doi:10.18632/oncotarget.9817)
Supplement: Supplementary file 1 [file oncotarget-07-42071-s001.pdf]

Presence of TMPRSS2-ERG is associated with alterations of the metabolic profile in human prostate cancer

Supplementary Materials

Supplementary Table S1: Sample distribution per patient in the main cohort classified as ERG_{low}, $ERG_{intermediate}$ and ERG_{high}

Patient	Benign	ERG_{low}	$ERG_{intermediate}$	ERG_{high}	Total
1	1	0	0	2	3
2	1	0	3	1	5
3	1	0	0	2	3
4	1	0	1	1	3
5	1	0	1	1	3
6	0	1	1	2	4
7	1	0	0	2	3
8	0	0	0	4	4
9	0	2	0	0	2
10	1	3	1	0	5
11	1	1	2	1	5
12	1	1	2	0	4
13	1	0	1	0	2
14	0	0	2	0	2
15	1	1	0	1	3
16	2	0	1	2	5
17	1	0	0	1	2
18	1	0	1	2	4
19	0	0	0	3	3
20	1	3	0	0	4
21	1	2	0	0	3
22	1	0	1	1	3
23	1	1	2	0	4
24	0	0	0	2	2
25	1	0	0	0	1
26	1	1	0	0	2
27	1	1	0	0	2
28	1	0	0	0	1
29	1	1	1	0	3
30	2	2	0	0	4
31	1	1	0	0	2
32	1	1	0	0	2

33	1	0	0	0	1
34	0	1	2	0	3
35	0	1	1	3	5
36	2	1	0	0	3
37	1	2	2	1	6
38	0	0	2	1	3
39	1	1	1	1	4
40	1	2	0	0	3
41	0	0	3	0	3
Total	34	30	31	34	129

Supplementary Table S2: Differences in levels of quantified metabolites in the main cohort comparing ERG_{high} samples to ERG_{low} samples

Metabolites	Benign	ERG_{low}	$ERG_{intermediate}$	ERG_{high}	ERG_{high} vs ERG_{low} *p*-values	ERG_{high} vs ERG_{low} *p*-values*	*p*-trend**	Corrected for stromal content	Corrected for cancer content	Corrected for benign epithelial content	Corrected for luminal space content
	n = 34	*n* = 30	*n* = 31	*n* = 34							
	Concentrations mmoles/kg wet weight, median (IQR)	Concentrations mmoles/kg wet weight, median (IQR)	Concentrations mmoles/kg wet weight, median (IQR)	Concentrations mmoles/kg wet weight, median (IQR)							
Alanine	1.74 (1.32 to 2.09)	2.18 (1.75 to 2.79)	2.16 (1.65 to 2.57)	2.48 (1.78 to 2.85)	0.711	0.866	0.611	0.876	0.929	0.744	0.666
Choline	0.45 (0.28 to 0.61)	0.99 (0.59 to 1.43)	1.01 (0.65 to 1.68)	1.23 (0.89 to 1.51)	0.126	0.29	0.116	0.096	0.102	0.043	0.038
Citrate	8.45 (4.62 to 11.85)	9.44 (5.56 to 14.68)	6.74 (3.94 to 10.34)	3.91 (2.59 to 7.20)	< 0.001	< 0.001	< 0.001	5.60E-06	2.20E-05	6.10E-05	5.80E-05
Creatine	2.51 (1.97 to 3.06)	2.27 (1.65 to 2.70)	2.10 (1.82 to 2.55)	2.00 (1.64 to 2.54)	0.953	1	0.546	0.913	0.817	0.666	0.729
Ethm	0 (0 to 0.18)	0 (0 to 0)	0 (0 to 0.15)	0.01 (0 to 0.31)	0.49	0.663	0.043	0.421	0.352	0.238	0.326
Glucose	0.78 (0.47 to 1.25)	0.15 (0.00 to 0.63)	0.00 (0.00 to 0.43)	0.00 (0.00 to 0.07)	0.008	0.061	0.007	0.012	0.021	0.014	6.40E-03
Glutamate	2.73 (2.42 to 3.53)	4.60 (4.02 to 6.11)	4.75 (3.55 to 6.88)	5.98 (4.26 to 7.79)	0.139	0.291	0.101	0.133	0.244	0.116	0.049
Glutamine	2.05 (1.58 to 2.35)	2.57 (2.27 to 3.41)	2.82 (2.14 to 3.50)	3.05 (2.61 to 3.70)	0.422	0.647	0.12	0.485	0.281	0.413	0.3
Glycine	1.55 (1.28 to 1.98)	1.99 (1.68 to 2.78)	2.51 (1.98 to 3.18)	2.90 (1.93 to 3.65)	0.023	0.115	0.008	5.70E-03	1.00E-02	6.10E-03	4.00E-03
GPC	0.41 (0.25 to 0.50)	0.76 (0.49 to 1.28)	0.73 (0.43 to 1.41)	0.90 (0.60 to 1.17)	1	1	0.865	0.72	0.563	0.823	0.653
GPE	0.23 (0 to 0.43)	0.17 (0 to 0.55)	0.03 (0 to 0.51)	0 (0 to 0.72)	0.999	1	0.94	0.913	0.862	0.909	0.831
Isoleucine	0.09 (0 to 0.12)	0.17 (0.03 to 0.24)	0.15 (0.06 to 0.29)	0.19 (0.11 to 0.31)	0.097	0.248	0.138	0.024	0.014	0.022	0.05
Lactate	13.93 (10.35 to 16.54)	21.92 (16.52 to 25.63)	20.52 (13.61to 26.41)	17.34 (15.69 to 22.01)	0.34	0.602	0.166	0.148	0.134	0.178	0.174
Leucine	0.24 (0.16 to 0.36)	0.38 (0.30 to 0.60)	0.46 (0.32 to 0.70)	0.51 (0.37 to 0.70)	0.715	0.866	0.168	0.746	0.788	0.598	0.668
Myo-inositol	9.06 (8.01 to 10.67)	10.03 (6.92 to 11.67)	9.13 (7.16 to 12.66)	8.97 (7.23 to 10.56)	0.49	0.663	0.294	0.373	0.431	0.454	0.475
PCh	0.32 (0.16 to 0.60)	0.61 (0.33 to 0.91)	0.63 (0.43 to 1.17)	0.96 (0.64 to 1.36)	0.067	0.248	0.005	0.017	0.027	0.026	0.018
PE	1.73 (1.09 to 2.59)	2.33 (1.46 to 3.51)	2.57 (1.67 to 4.14)	2.86 (2.33 to 3.79)	0.087	0.248	0.032	0.02	0.029	0.042	0.045
Putrescine	0.44 (0 to 1.34)	0.12 (0 to 0.67)	0.06 (0 to 0.30)	0 (0 to 0.10)	0.025	0.115	0.003	2.20E-03	4.00E-03	7.70E-03	6.40E-03
Scyllo-inositol	0.33 (0.25 to 0.56)	0.39 (0.31 to 0.59)	0.50 (0.37 to 0.63)	0.47 (0.37 to 0.61)	0.087	0.248	0.438	4.50E-03	5.50E-03	0.029	0.067
Spermine	1.69 (0.90 to 2.71)	2.10 (1.20 to 3.19)	1.23 (0.79 to 2.02)	0.89 (0.45 to 1.40)	< 0.001	< 0.001	< 0.001	7.20E-06	3.10E-05	9.90E-05	7.00E-05
Succinate	0.37 (0.30 to 0.47)	0.60 (0.43 to 0 .74)	0.62 (0.44 to 0.89)	0.63 (0.49 to 0.88)	0.412	0.647	0.446	0.401	0.433	0.247	0.181
Taurine	5.74 (3.98 to 6.27)	5.39 (3.90 to 6.96)	4.84 (3.48 to 7.09)	4.26 (3.28 to 5.54)	0.168	0.322	0.097	0.117	0.167	0.14	0.071
Valine	0.21 (0.18 to 0.29)	0.35 (0.27 to 0.50)	0.39 (0.22 to 0.49)	0.39 (0.26 to 0.53)	0.972	1	0.666	0.975	0.983	0.847	0.999

Ethm: Ethanolamine, GPC: Glycerophosphocholine, GPE: Glycerophosphoethanolamine, PCh: Phosphocholine, PE: Phosphoethanolamine.
*Benjamini-Hochberg adjusted values, **trend across increasing ERG groups (not including benign samples).

Supplementary Table S3: Differences in levels of quantified metabolites in the main cohort, comparing all ERG groups to benign samples and ERG_{low} to $ERG_{intermediate}$ and $ERG_{intermediate}$ to ERG_{high}

Metabolites	Benign vs ERG_{low}	Benign vs $ERG_{intermediate}$	Benign vs ERG_{high}	ERG_{low} vs ERG_{int}	ERG_{int} vs ERG_{high}
	p-value	*p*-value	*p*-value	*p*-value	*p*-value
**Alanine**	2.80E–03	0.044	5.30E–03	0.728	0.779
**Choline**	9.70E–06	1.70E–07	8.70E–09	0.035	0.687
**Citrate**	0.334	0.099	1.30E–04	0.015	0.19
**Creatine**	0.692	0.995	0.342	0.282	0.518
**Ethm**	0.161	0.476	0.739	0.356	0.633
**Glucose**	5.40E–06	4.50E–11	1.40E–14	0.094	0.374
**Glutamate**	4.30E–09	7.00E–10	4.50E–13	0.15	0.239
**Glutamine**	6.80E–04	1.90E–03	1.30E–06	0.332	0.381
**Glycine**	6.30E–03	1.60E–04	2.10E–07	0.091	0.39
**GPC**	1.10E–09	2.20E–07	1.50E–06	0.32	0.316
**GPE**	0.986	0.604	0.84	0.601	0.945
**Isoleucine**	0.109	0.015	3.60E–04	0.254	0.177
**Lactate**	8.70E–08	6.00E–06	1.00E–04	0.881	0.386
**Leucine**	2.50E–04	2.80E–07	1.60E–05	0.719	0.847
**Myo–inositol**	0.286	0.222	0.751	0.927	0.327
**PCh**	9.00E–03	1.80E–06	1.00E–08	0.077	0.273
**PE**	1.60E–03	4.40E–06	2.90E–08	0.238	0.501
**Putrescine**	0.261	7.80E–03	5.70E–05	0.03	0.939
**Scyllo–inositol**	0.372	0.608	0.688	0.963	0.492
**Spermine**	0.118	0.201	3.80E–05	0.035	0.086
**Succinate**	0.016	0.019	4.70E–06	0.243	0.838
**Taurine**	0.796	0.489	0.104	0.827	0.035
**Valine**	0.065	0.152	0.02	0.764	0.359

Ethm: Ethanolamine, GPC: Glycerophosphocholine, GPE: Glycerophosphoethanolamine.
GSH: Glutathione, PCh:Phosphocholine, PE: Phosphoethanolamine.

Supplementary Table S4: Differences in levels of quantified metabolites in the validation cohort comparing TMPRSS2–ERG positive and negative patients

Metabolites	TMPRSS2:ERG negative $n = 33$ Concentrations mmoles/kg wet weight (IQR)	TMPRSS2:ERG positive $n = 7$ Concentrations mmoles/kg wet weight (IQR)	*p*-value	*p*-value*
**Alanine**	2.51 (2.07 to 2.88)	2.13 (1.59 to 2.51)	0.202	0.721
**Ascorbate**	0.06 (0.00 to 0.06)	0.08 (0.00 to 0.22)	0.487	0.84
**Choline**	0.96 (0.79 to 1.19)	0.79 (0.40 to 0.99)	0.148	0.617
**Citrate**	6.74 (3.88 to 8.74)	3.05 (1.25 to 4.69)	0.013	0.263
**Creatine**	3.01 (2.61 to 3.51)	2.69 (2.14 to 3.66)	0.293	0.775
**Ethm**	0.22 (0.00 to 0.36)	0.00 (0.00 to 0.00)	0.088	0.55
**Glucose**	0.39 (0.00 to 0.77)	0.55 (0.00 to 1.56)	0.805	0.957
**Glutamate**	4.08 (3.12 to 4.62)	3.94 (3.45 to 4.64)	0.832	0.957
**Glutamine**	2.09 (1.61 to 2.47)	2.09 (1.78 to 2.55)	0.919	0.957
**Glycine**	2.37 (1.92 to 2.86)	2.41 (1.54 to 2.64)	0.857	0.957
**GPC**	0.90 (0.52 to 1.14)	0.85 (0.48 to 1.21)	0.905	0.957
**GPE**	0.13 (0.00 to 0.16)	0.18 (0.00 to 0.07)	0.099	0.55
**GSH**	0.84 (0.74 to 1.04)	0.86 (0.64 to 1.13)	0.898	0.957
**Isoleucine**	0.17 (0.09 to 0.25)	0.13 (0.08 to 0.16)	0.413	0.794
**Lactate**	16.01 (12.74 to 18.12)	15.92 (12.34 to 20.40)	0.959	0.959
**Leucine**	0.36 (0.22 to 0.47)	0.40 (0.27 to 0.38)	0.571	0.892
**Myo–inositol**	8.82 (7.31 to 10.05)	8.10 (7.09 to 8.90)	0.504	0.84
**PCh**	0.62 (0.31 to 0.81)	0.85 (0.63 to 0.98)	0.11	0.55
**PE**	2.34 (1.82 to 2.78)	2.68 (2.18 to 2.80)	0.282	0.775
**Putrescine**	0.17 (0.00 to 0.27)	0.06 (0.00 to 0.00)	0.348	0.791
**Scyllo–inositol**	0.60 (0.38 to 0.78)	0.48 (0.34 to 0.47)	0.381	0.794
**Spermine**	0.69 (0.38 to 0.81)	0.31 (0.10 to 0.52)	**0.021**	0.263
**Succinate**	0.54 (0.40 to 0.69)	0.46 (0.37 to 0.57)	0.31	0.775
**Taurine**	5.81 (4.83 to 7.06)	5.66 (5.16 to 6.80)	0.869	0.957
**Valine**	0.48 (0.41 to 0.50)	0.46 (0.39 to 0.49)	0.81	0.957

Ethm: Ethanolamine, GPC: Glycerophosphocholine, GPE: Glycerophosphoethanolamine, GSH: Glutathione, PCh: Phosphocholine, PE: Phosphoethanolamine *Benjamini-Hochberg corrected for multiple testing.

Supplementary Table S5: Differences in expression levels of key metabolic genes comparing ERG_{high} and ERG_{low} in the main cohort, including estimated means and adjusted for benign epithelia, stroma, cancer and luminal space

Gene	Estimated/predicted means med 95 % CI		*p*-value	*p*-value*	Adjusted for benign epithelial content	Adjusted for stromal content	Adjusted for cancer content	Adjusted for luminal space content
	ERG_{low}	ERG_{high}						
**ACACA**	9.81 (9.67, 9.94)	10.10 (9.97, 10.24)	9.80E–04	0.003	2.50E–03	1.50E–03	3.10E–03	4.70E–04
**ACO1**	9.71 (9.61, 9.82)	9.40 (9.30, 9.51)	2.50E–06	6.09E–05	6.30E–06	6.00E–06	1.40E–05	2.60E–06
**ACO2**	8.06 (7.97, 8.15)	7.93 (7.85, 8.02)	0.036	0.065	0.031	0.013	0.014	0.046
**ACSL1**			0.683	0.683	0.715	0.593	0.606	0.64
**ACSL3**	10.78 (10.59, 10.97)	11.07 (10.88, 11.26)	5.80E–03	0.014	1.40E–03	4.80E–03	1.50E–03	8.80E–03
**ACSL4**	7.08 (6.95, 7.22)	6.82 (6.69, 6.95)	3.80E–03	0.010	6.90E–03	6.20E–03	0.012	4.00E–03
**ACSL5**	6.20 (5.85, 6.55)	6.67 (6.33, 7.01)	0.045	0.073	0.021	0.035	0.018	0.045
**ACSL6**			0.264	0.320	0.312	0.209	0.247	0.171
**AGXT1**			0.155	0.208	0.193	0.215	0.255	0.139
**ALDOA**	9.33 (9.15, 9.50)	8.79 (8.62, 8.96)	1.40E–05	1.89E–04	2.40E–05	2.20E–05	4.80E–05	1.90E–05
**ALDOB**	6.03 (5.85, 6.20)	6.37 (6.20, 6.55)	3.40E–03	0.009	3.40E–03	6.50E–03	8.40E–03	9.70E–04
**ALDOC**			0.38	0.420	0.43	0.546	0.636	0.311
**AMD1**	6.93 (6.73, 7.13)	7.28 (7.08, 7.48)	3.40E–03	0.009	1.70E–03	4.50E–03	2.80E–03	2.70E–03
**CHKA**	8.10 (7.99, 8.21)	8.42 (8.32, 8.53)	1.60E–05	1.89E–04	2.70E–05	2.00E–05	3.10E–05	2.50E–05
**CS**			0.148	0.203	0.175	0.144	0.161	0.12
**DLD**	7.83 (7.71, 7.94)	7.59 (7.47, 7.71)	3.40E–04	0.001	6.10E–04	4.60E–04	7.70E–04	8.40E–04
**DLST**			0.132	0.189	0.115	0.074	0.054	0.14
**ENO1**	11.39 (11.29, 11.49)	11.64 (11.55, 11.74)	8.10E–05	4.78E–04	1.70E–04	1.20E–04	2.10E–04	9.90E–05
**FASN**	11.62 (11.36, 11.87)	11.98 (11.72, 12.23)	0.015	0.030	0.014	0.017	0.015	6.80E–03
**FH**			0.549	0.567	0.58	0.365	0.361	0.853
**G6PD**	6.95 (6.86, 7.04)	7.08 (6.99, 7.17)	0.033	0.061	0.037	0.044	0.053	0.028
**GAPDH**			0.285	0.339	0.33	0.392	0.446	0.181
**GPI**			0.652	0.663	0.75	0.818	0.906	0.559
**HK1**			0.06	0.095	0.057	0.033	0.033	0.063
**HK2**			0.241	0.298	0.053	0.121	0.025	0.344
**IDH1**			0.324	0.378	0.415	0.323	0.394	0.157
**IDH2**			0.191	0.241	0.164	0.231	0.215	0.15
**IDH3A**			0.338	0.380	0.322	0.281	0.253	0.404
**IDH3B**	8.92 (8.84, 8.99)	8.74 (8.67, 8.82)	3.70E–04	0.001	4.40E–04	5.70E–04	6.90E–04	4.10E–04
**IDH3G**			0.119	0.179	0.179	0.17	0.279	0.081
**LDHA**			0.178	0.229	0.223	0.258	0.315	0.239
**LDHB**	9.41 (9.13, 9.68)	8.77 (8.50, 9.05)	1.90E–04	9.21E–04	4.00E–04	2.80E–04	5.20E–04	3.00E–06
**MDH1**	10.33 (10.22, 10.45)	10.19 (10.07, 10.30)	0.041	0.068	0.036	0.082	0.079	7.30E–03
**MDH2**	11.93 (11.86, 12.00)	12.10 (12.03, 12.16)	4.00E–05	3.60E–04	2.60E–05	6.90E–05	6.10E–05	2.80E–05

**NPY**	10.93 (10.18, 11.68)	13.24 (12.50, 13.98)	2.90E–06	6.09E–05	1.60E–06	3.30E–06	2.10E–06	6.10E–06
**OAZ**			0.17	0.223	0.177	0.174	0.091	0.587
**ODC**	10.75 (10.49, 11.01)	11.12 (10.85, 11.38)	0.012	0.024	0.013	0.032	0.04	7.50E–03
**OGDH**			0.075	0.115	0.056	0.092	0.079	0.071
**OGDHL**	6.13 (5.94, 6.32)	7.48 (7.29, 7.66)	7.70E–29	4.85E–27	2.50E–33	2.10E–28	2.80E–30	5.90E–28
**PDHA**	8.55 (8.47, 8.62)	8.69 (8.62, 8.76)	7.90E–03	0.018	0.011	9.70E–03	0.014	8.90E–03
**PDHB**			0.448	0.487	0.479	0.384	0.418	0.424
**PGD**	8.41 (8.29, 8.53)	8.66 (8.54, 8.77)	2.80E–04	0.001	4.30E–04	6.20E–04	1.20E–03	5.70E–04
**PGK1**	10.36 (10.23, 10.50)	10.04 (9.91, 10.17)	7.70E–05	4.78E–04	1.00E–04	6.10E–05	9.50E–05	3.40E–05
**PGLS**	8.69 (8.58, 8.79)	8.94 (8.84, 9.04)	9.10E–05	4.78E–04	1.80E–04	2.20E–04	4.40E–04	1.70E–05
**PGLS**	8.69 (8.59, 8.79)	8.94 (8.84, 9.04)	9.10E–05	4.78E–04	1.80E–04	2.20E–04	4.40E–04	1.70E–05
**PGM1**	9.56 (9.40, 9.73)	9.19 (9.03, 9.36)	1.10E–03	0.003	1.80E–03	1.70E–03	3.40E–03	1.20E–03
**PKM2**	9.20 (9.09, 9.31)	9.36 (9.26, 9.47)	0.027	0.052	0.027	0.028	0.027	0.025
**RBKS**	6.82 (6.73, 6.91)	7.05 (6.97, 7.14)	2.50E–04	0.001	6.10E–04	3.50E–04	8.10E–04	3.20E–04
**RPE**			0.335	0.380	0.332	0.244	0.24	0.443
**RPIA**	7.44 (7.30, 7.58)	7.62 (7.49, 7.76)	0.04	0.068	0.051	0.06	0.075	4.40E–03
**SAT1**	12.27 (12.12, 12.43)	12.64 (12.49, 12.80)	4.00E–04	0.001	1.30E–04	2.40E–04	9.80E–05	1.30E–03
**SDHA**	8.67 (8.59, 8.74)	8.55 (8.47, 8.62)	0.012	0.024	0.013	0.016	0.017	0.015
**SDHB**			0.548	0.567	0.492	0.528	0.584	0.609
**SDHC**			0.132	0.189	0.139	0.287	0.161	0.216
**SDHD**	7.59 (7.44, 7.75)	7.33 (7.17, 7.48)	0.011	0.024	0.012	0.013	0.311	6.50E–03
**SHMT1**	7.01 (6.9, 7.2)	7.31 (7.16, 7.46)	0.003	0.009	0.003	0.001	0.002	2.00E–03
**SMOX**	7.15 (6.86, 7.43)	7.75 (7.47, 8.03)	1.80E–05	1.89E–04	3.00E–05	2.50E–05	4.00E–05	1.10E–06
**SMS**	9.73 (9.46, 10.00)	9.37 (9.10, 9.64)	0.041	0.068	0.019	0.011	4.60E–03	0.067
**SRM**	8.06 (7.94, 8.19)	8.30 (8.17, 8.42)	6.20E–03	0.014	0.011	9.00E–03	0.015	5.20E–03
**SUCLG**			0.148	0.203	0.165	0.137	0.142	0.198
**TALDO1**			0.506	0.540	0.637	0.461	0.552	0.473
**TKT**	9.46 (9.31, 9.61)	9.86 (9.71, 10.01)	4.80E–05	3.78E–04	1.10E–04	1.00E–04	2.50E–04	6.20E–06
**TPI1**	9.69 (9.52, 9.86)	9.30 (9.14, 9.47)	4.50E–04	0.001	7.60E–04	7.00E–04	1.10E–03	7.00E–04

ACACA: Acetyl-CoA carboxylase alpha, ACO1: Aconitase 1, ACO2: Aconitase 2, ACSL1: Acyl-CoA synthetase 1, ACSL3: Acyl-CoA synthetase 3, ACSL4: Acyl-CoA synthetase 4,ACSL5: Acyl-CoA synthetase 5, ACSL6: Acyl-CoA synthetase 6, AGXT1: Alanine-glyoxylate aminotransferase 1, ALDOA: Aldolase A, ALDOB: Aldolase B, ALDOC: Aldolase C,AMD1: Adenosylmethionine decarboxylase, CHKA: Choline kinase alpha, CS: Citrate synthase, DLD: Dihydrolipoamide dehydrogenase, DLST: Dihydrolipoamides–succinyltransferase, ENO1: Enolase 1, FASN: Fatty acid synthase, FH: Fumarate hydratase, G6PD: Glucose-6-phosphate dehydrogenase, GAPDH: Glyceraldehyde-3-phosphatedehydrogenase, GPI: Glucose-6-phosphate isomerase, HK1: Hexokinase 1, HK2: Hexokinase 2, IDH1: Isocitrate dehydrogenase 1, IDH2: Isocitrate dehydrogenase 2, IDH3A:Isocitrate dehydrogenase 3A, IDH3B: Isocitrate dehydrogenase 3B, IDH3G: Isocitrate dehydrogenase 3G, MDH1: Malate dehydrogenase 1, MDH2: Malate dehydrogenase 2, NPY:Neuropeptide Y, OAZ: Ornithine decarboxylase antizyme, ODC: Ornithine decarboxylase, OGDH: Oxoglutarate (alpha–ketoglutarate) dehydrogenase, OGDHL: Oxoglutarate dehydrogenase-like, PDHA: Pyruvate dehydrogenase alpha, PDHB: Pyruvate dehydrogenase beta, PGK1: Phosphoglycerate kinase, PGD: Phosphogluconate dehydrogenase, PGLS: 6-phosphogluconolactonase, PGM1: Phosphoglucomutase, PKM2: Pyruvate kinase, RBKS: Ribokinase, RPE: Ribulose-5-phosphate-3-epimerase, RPIA: Ribose 5-phosphate isomerase, SAT1: Spermidine/spermine-N1-acetyltransferase 1, SDHA: Succinate dehydrogenase complex, subunit A, SDHB: Succinate dehydrogenase complex, subunit B, SDHD: Succinate dehydrogenase complex, subunit D, SHMT1: Serine hydroxymethyltransferase 1, SMOX: Spermine oxidase, SMS: Spermine synthase, SRM: Spermidine synthase, SUCLG: Succinyl-CoA ligase, TALDO1: Transaldolase 1, TKT: Transketolase, TPI1: Triosephosphate isomerase 1. Since the gene expression values were log2 transformed, a difference in expression by one unit corresponds to a twofold mean change in probe intensities. *Benjamini–Hochberg corrected.

Supplementary Table S6: Differences in expression levels of key metabolic genes comparing ERG_{high} and ERG_{low} in low Gleason (Gleason ≤ 3 + 4) samples in the main cohort, including estimated means and adjusted for benign epithelia, stroma, cancer and luminal space

Gene	Estimated/predicted means med 95 % CI		*p*-value	*p*-value*	Adjusted for benign epithelial content	Adjusted for stromal content	Adjusted for cancer content	Adjusted for luminal space content
	ERG_{low}	ERG_{high}						
**ACACA**	9.81 (9.64, 9.98)	10.19 (10.04, 10.33)	2.90E–04	0.001	0.247	0.55	0.355	0.22
**ACO1**	9.70 (9.58, 9.83)	9.47 (9.36, 9.58)	4.20E–03	0.010	0.016	0.026	8.90E–03	0.041
**ACO2**	8.12 (8.01, 8.23)	7.91 (7.81, 8.00)	2.80E–03	0.007	0.016	7.60E–04	4.40E–03	0.016
**ACSL1**	9.30 (8.99, 9.60)	8.86 (8.59, 9.13)	0.017	0.029	0.026	0.072	0.078	0.028
**ACSL3**			0.294	0.331	1.06E–01	0.062	6.00E–02	8.70E–02
**ACSL4**	7.18 (7.01, 7.35)	6.76 (6.62, 6.91)	1.80E–04	7.56E–04	0.552	0.647	0.615	0.617
**ACSL5**			0.248	0.284	6.40E–05	0.029	1.90E–04	2.10E–04
**ACSL6**			0.826	0.853	0.14	0.136	0.22	0.488
**AGXT1**			0.951	0.957	3.70E–07	4.40E–06	6.30E–07	4.30E–07
**ALDOA**	9.39 (9.14, 9.63)	8.74 (8.53, 8.94)	5.80E–05	3.32E–04	8.90E–03	0.022	0.015	0.015
**ALDOB**	6.06 (5.82, 6.30)	6.45 (6.25, 6.65)	0.014	0.025	2.00E–04	2.80E–04	5.20E–05	2.00E–05
**ALDOC**	7.43384 (7.29, 7.58)	7.05 (6.92, 7.19)	3.30E–06	2.97E–05	0.526	0.563	0.62	0.497
**AMD1**	6.93 (6.66, 7.21)	7.35 (7.11, 7.59)	0.013	0.024	3.70E–05	3.70E–05	2.40E–05	4.70E–05
**CHKA**			0.129	0.159	4.30E–03	6.20E–05	6.30E–05	1.10E–04
**CS**			0.638	0.681	4.10E–03	9.70E–03	5.50E–03	5.50E–03
**DLD**	7.89 (7.73, 8.04)	7.55 (7.41, 7.70)	2.00E–06	2.10E–05	1.40E–04	4.70E–04	1.70E–04	8.80E–05
**DLST**			0.321	0.355	1.10E–04	2.40E–13	3.70E–04	2.50E–11
**ENO1**	11.29 (11.15, 11.43)	11.63 (11.51, 11.74)	2.60E–04	0.001	1.50E–06	1.50E–11	8.70E–03	1.50E–06
**FASN**			0.066	0.099	1.30E–04	2.10E–03	2.90E–04	2.50E–04
**FH**			0.957	0.957	2.10E–03	3.20E–03	3.50E–03	3.50E–03
**G6PD**			0.111	0.143	0.767	0.319	0.564	0.942
**GAPDH**	11.36 (11.22, 11.51)	11.59 (11.47, 11.72)	7.00E–03	0.015	7.90E–04	6.30E–07	3.40E–03	2.60E–08
**GPI**			0.406	0.441	0.15	0.02	0.049	0.06
**HK1**	5.99 (5.86, 6.12)	5.78 (5.67, 5.90)	0.015	0.026	0.03	4.00E–03	4.00E–03	4.40E–04
**HK2**			0.077	0.108	2.30E–03	5.40E–03	6.40E–03	1.30E–03
**IDH1**	9.06 (8.76, 9.37)	8.64 (8.37, 8.91)	0.02	0.033	0.011	0.011	0.012	6.90E–03
**IDH2**	9.03 (8.87, 9.18)	9.23 (9.09, 9.36)	0.038	0.058	0.081	0.144	0.104	0.108
**IDH3A**			0.184	0.223	3.50E–03	6.10E–06	1.50E–03	3.40E–05
**IDH3B**	8.91 (8.82, 9.01)	8.75 (8.67, 8.84)	9.80E–03	0.019	0.017	0.011	0.091	2.10E–03
**IDH3G**	8.43 (8.31, 8.54)	8.74 (8.63, 8.84)	2.60E–08	4.10E–07	0.032	7.20E–03	0.019	0.017
**LDHA**			0.072	0.105	3.60E–04	0.073	0.027	0.045
**LDHB**	9.44 (9.05, 9.83)	8.83 (8.49, 9.17)	0.012	0.023	0.039	0.14	0.227	0.028
**MDH1**	10.34 (10.18, 10.50)	10.15 (10.01, 10.29)	0.038	0.058	0.227	0.023	0.077	0.068
**MDH2**	11.89 (11.80, 11.99)	12.14 (12.06, 12.22)	4.10E–05	2.58E–04	3.60E–05	2.30E–04	4.50E–05	6.00E–05
**NPY**	10.41 (9.34, 11.48)	13.24 (12.33, 14.15)	6.40E–05	3.36E–04	4.70E–04	8.20E–07	3.00E–03	3.70E–06
**OAZ**	6.57 (6.33, 6.81)	6.38 (6.15, 6.62)	3.10E–03	0.008	0.123	0.029	0.026	0.08
**ODC**	10.68 (10.28, 11.09)	11.22 (10.86, 11.58)	0.028	0.045	9.10E–23	5.50E–23	2.50E–21	1.80E–24
**OGDH**	8.35 (8.24, 8.47)	8.54 (8.44, 8.64)	8.60E–03	0.017	0.062	0.055	0.044	0.07
**OGDHL**	5.95 (5.70, 6.21)	7.62 (7.41, 7.84)	3.40E–24	2.14E–22	4.80E–04	9.40E–04	2.40E–03	6.30E–05
**PDHA**			0.096	0.129	4.60E–07	2.50E–12	4.60E–07	4.70E–13
**PDHB**			0.107	0.140	2.20E–04	1.30E–03	4.40E–04	4.40E–04
**PGD**	8.38 (8.23, 8.52)	8.70 (8.59, 8.82)	3.30E–04	0.001	3.60E–04	3.40E–04	2.40E–04	3.00E–04

**PGK1**	10.36 (10.15, 10.57)	9.97 (9.79, 10.16)	4.40E–03	0.010	0.133	0.164	0.107	0.186
**PGLS**	8.69 (8.54, 8.84)	8.97 (8.84, 9.10)	2.10E–03	0.006	0.017	0.019	0.045	0.015
**PGLS**	8.69 (8.54, 8.84)	8.97 (8.84, 9.10)	2.10E–03	0.006	0.37	0.974	0.732	0.407
**PGM1**	9.64 (9.41, 9.88)	9.15 (8.95, 9.35)	1.80E–03	0.005	0.123	0.211	0.12	0.1
**PKM2**	9.06 (8.95, 9.18)	9.43 (9.32, 9.54)	2.90E–13	9.14E–12	4.10E–03	0.01	7.30E–03	3.80E–03
**RBKS**	6.73 (6.60, 6.86)	7.05 (6.94, 7.16)	3.80E–04	0.001	0.062	0.087	0.079	0.063
**RPE**			0.755	0.793	5.90E–04	1.90E–04	3.10E–03	3.90E–05
**RPIA**			0.241	0.281	9.50E–04	1.90E–03	3.60E–03	2.00E–03
**SAT1**	12.05 (11.82, 12.27)	12.64 (12.45, 12.83)	9.10E–05	4.10E–04	0.123	0.22	0.16	0.116
**SDHA**	8.70 (8.63, 8.78)	8.53 (8.46, 8.60)	3.30E–05	2.31E–04	0.966	0.901	0.92	0.955
**SDHB**			0.206	0.245	9.80E–03	0.148	0.25	0.012
**SDHC**	7.75 (7.53, 7.96)	7.30 (7.11, 7.49)	9.00E–04	0.003	0.951	0.703	0.716	0.957
**SDHD**	7.72 (7.52, 7.92)	7.19 (7.01, 7.37)	8.90E–06	7.01E–05	0.814	0.931	0.873	0.915
**SHMT1**			0.077	0.108	2.30E–03	5.40E–03	6.40E–03	1.30E–03
**SMOX**	7.21 (6.92, 7.51)	8.08 (7.80, 8.36)	1.40E–11	2.94E–10	3.10E–03	6.50E–04	1.80E–03	2.90E–03
**SMS**			0.094	0.129	1.10E–03	5.00E–03	1.70E–03	2.10E–03
**SRM**	8.06 (7.90, 8.21)	8.35 (8.22, 8.47)	5.00E–03	0.011	9.50E–03	0.017	0.011	0.01
**SUCLG**	9.62 (9.49, 9.75)	9.32 (9.20, 9.44)	7.40E–05	3.59E–04	0.012	0.014	0.011	0.017
**TALDO1**			0.122	0.154	0.221	0.383	0.4	0.228
**TKT**	9.30 (9.07, 9.52)	10.00 (9.80, 10.19)	8.10E–07	1.02E–05	0.1	0.36	0.177	0.447
**TPI1**	9.73 (9.50, 9.95)	9.29 (9.10, 9.48)	3.30E–03	0.008	0.088	0.019	0.092	0.01

ACACA: Acetyl-CoA carboxylase alpha, ACO1: Aconitase 1, ACO2: Aconitase 2, ACSL1: Acyl-CoA synthetase 1, ACSL3: Acyl-CoA synthetase 3, ACSL4: Acyl-CoA synthetase 4,ACSL5: Acyl-CoA synthetase 5, ACSL6: Acyl-CoA synthetase 6, AGXT1: Alanine-glyoxylate aminotransferase 1, ALDOA: Aldolase A, ALDOB: Aldolase B, ALDOC: Aldolase C,AMD1: Adenosylmethionine decarboxylase, CHKA: Choline kinase alpha, CS: Citrate synthase, DLD: Dihydrolipoamide dehydrogenase, DLST: Dihydrolipoamides-succinyltransferase, ENO1: Enolase 1, FASN: Fatty acid synthase, FH: Fumarate hydratase, G6PD: Glucose-6-phosphate dehydrogenase, GAPDH: Glyceraldehyde-3-phosphatedehydrogenase, GPI: Glucose-6-phosphate isomerase, HK1: Hexokinase 1, HK2: Hexokinase 2, IDH1: Isocitrate dehydrogenase 1, IDH2: Isocitrate dehydrogenase 2, IDH3A:Isocitrate dehydrogenase 3A, IDH3B: Isocitrate dehydrogenase 3B, IDH3G: Isocitrate dehydrogenase 3G, MDH1: Malate dehydrogenase 1, MDH2: Malate dehydrogenase 2, NPY:Neuropeptide Y, OAZ: Ornithine decarboxylase antizyme, ODC: Ornithine decarboxylase, OGDH: Oxoglutarate (alpha-ketoglutarate) dehydrogenase, OGDHL: Oxoglutarate dehydrogenase-like, PDHA: Pyruvate dehydrogenase alpha, PDHB: Pyruvate dehydrogenase beta, PGK1: Phosphoglycerate kinase, PGD: Phosphogluconate dehydrogenase, PGLS: 6-phosphogluconolactonase, PGM1: Phosphoglucomutase, PKM2: Pyruvate kinase, RBKS: Ribokinase, RPE: Ribulose-5-phosphate-3-epimerase, RPIA: Ribose 5-phosphate isomerase, SAT1: Spermidine/spermine-N1-acetyltransferase 1, SDHA: Succinate dehydrogenase complex, subunit A, SDHB: Succinate dehydrogenase complex, subunit B, SDHD: Succinate dehydrogenase complex, subunit D, SHMT1: Serine hydroxymethyltransferase 1, SMOX: Spermine oxidase, SMS: Spermine synthase, SRM: Spermidine synthase, SUCLG: Succinyl-CoA ligase, TALDO1: Transaldolase 1, TKT: Transketolase, TPI1: Triosephosphate isomerase 1. Since the gene expression values were log2 transformed, a difference inexpression by one unit corresponds to a twofold mean change in probe intensities. *Benjamini-Hochberg corrected.

Supplementary Table S7: Differences in expression levels of key metabolic genes comparing ERG$_{high}$ and ERGl$_{ow}$ in high Gleason (Gleason ≥ 4 + 3) samples in the main cohort, including estimated means and adjusted for benign epithelia, stroma, cancer and luminal space

Gene	Estimated/predicted means med 95 % CI		*p*-value	*p*-value*	Adjusted for benign epithelial content	Adjusted for stromal content	Adjusted for cancer content	Adjusted for luminal space content
	ERG$_{low}$	ERG$_{high}$						
**ACACA**			0.054	0.144	0.741	0.55	0.794	0.431
**ACO1**	9.68 (9.52, 9.84)	9.37 (9.21, 9.52)	1.10E–03	0.023	0.402	0.699	0.395	0.708
**ACO2**			0.253	0.349	0.247	0.01	0.047	5.00E–03
**ACSL1**			0.444	0.519	0.665	0.418	0.488	0.386
**ACSL3**	10.55 (10.32, 10.78)	10.89 (10.66, 11.11)	0.017	0.082	0.048	0.094	0.107	0.036
**ACSL4**			0.201	0.288	0.247	0.01	0.047	5.00E–03
**ACSL5**			0.055	0.144	0.277	0.395	0.365	0.363
**ACSL6**			0.099	0.164	0.94	0.837	0.769	0.964
**AGXT1**			0.062	0.147	0.143	0.06	0.16	0.998
**ALDOA**	9.27(9.03, 9.51)	8.88 (8.63, 9.12	0.022	0.089	0.273	0.25	0.238	0.175
**ALDOB**			0.079	0.158	0.133	0.633	0.204	0.829
**ALDOC**			0.382	0.491	0.167	0.191	0.17	0.158
**AMD1**			0.085	0.158	0.932	0.738	0.632	0.95
**CHKA**	8.96 (8.83, 9.09)	9.44 (9.30, 9.57)	6.60E–07	2.08E–05	0.015	1.20E–03	2.90E–03	2.10E–03
**CS**			0.255	0.349	0.444	0.063	0.432	0.055
**DLD**			0.088	0.158	7.00E–03	1.20E–03	6.60E–04	0.025
**DLST**	6.89 (6.70, 7.07)	7.28 (7.09, 7.46)	1.50E–03	0.024	0.539	0.433	0.57	0.44
**ENO1**	11.47 (11.35, 11.59)	11.66 (11.53, 11.78)	0.024	0.089	5.80E–04	7.20E–04	1.70E–04	1.30E–04
**FASN**			0.115	0.186	1.30E–10	1.20E–10	4.60E–11	1.10E–10
**FH**			0.596	0.636	0.891	0.174	0.383	0.123
**G6PD**			0.194	0.284	0.468	0.123	0.309	0.228
**GAPDH**			0.531	0.589	0.035	0.018	0.013	0.01
**GPI**			0.533	0.589	9.40E–03	6.80E–03	0.011	1.60E–03
**HK1**			0.394	0.496	0.18	0.081	0.143	7.60E–03
**HK2**			0.453	0.519	0.774	0.896	0.771	0.931
**IDH1**			0.43	0.519	0.062	0.188	0.155	0.239
**IDH2**			0.063	0.147	0.167	0.448	0.27	0.483
**IDH3A**			0.093	0.160	8.40E–03	3.30E–03	2.90E–03	3.30E–03
**IDH3B**	8.92 (8.81, 9.03)	8.70 (8.59, 8.81)	4.30E–03	0.039	1.10E–03	0.057	0.011	0.042
**IDH3G**			0.592	0.636	0.012	0.064	0.014	0.102
**LDHA**			0.307	0.403	0.733	0.03	0.118	0.041
**LDHB**	9.39 (9.02, 9.75)	8.77 (8.40, 9.13)	0.011	0.063	0.034	0.092	0.205	6.20E–03
**MDH1**			0.903	0.918	0.308	0.083	0.179	0.029
**MDH2**			0.077	0.158	0.023	0.081	0.04	0.13
**NPY**	11.48 (10.49, 12.48)	13.35 (12.36, 14.34)	3.80E–03	0.039	0.888	0.156	0.544	0.285
**OAZ**	5.30 (5.12, 5.48)	4.92 (4.75, 5.10)	3.20E–03	0.039	0.012	0.018	0.014	0.016
**ODC**			0.084	0.158	0.106	0.011	0.03	1.10E–03

**OGDH**			0.805	0.831	0.964	0.798	0.755	0.626
**OGDHL**	6.33 (6.06, 6.60)	7.32 (7.06, 7.59)	1.70E–10	1.07E–08	0.747	0.053	0.135	0.038
**PDHA**	5.84 (5.74, 5.95)	5.66 (5.55, 5.77)	0.019	0.086	3.20E–03	6.80E–05	1.90E–04	1.60E–04
**PDHB**			0.951	0.951	0.09	0.033	0.062	0.024
**PGD**			0.142	0.224	0.069	0.02	0.016	0.012
**PGK1**	8.21 (8.05, 8.37)	8.01 (7.85, 8.17)	0.032	0.099	0.489	0.595	0.444	0.91
**PGLS**	8.68 (8.54, 8.81)	8.90 (8.77, 9.03)	1.00E–02	0.063	0.14	0.056	0.079	0.119
**PGLS**	8.68 (8.54, 8.81)	8.90 (8.77, 9.03)	1.00E–02	0.063	0.195	0.013	0.079	0.034
**PGM1**			0.094	0.160	0.085	0.097	0.162	0.039
**PKM2**			0.445	0.519	0.088	0.026	0.042	0.042
**RBKS**	6.89 (6.78, 7.00)	7.06 (6.94, 7.17)	0.037	0.106	0.717	0.625	0.752	0.347
**RPE**			0.194	0.284	0.013	0.037	0.023	4.20E–03
**RPIA**			0.08	0.158	0.678	0.518	0.692	0.314
**SAT1**	12.38 (12.18, 12.58)	12.66 (12.46, 12.85)	0.033	0.099	0.761	0.133	0.418	0.252
**SDHA**			0.061	0.147	0.496	0.48	0.921	0.452
**SDHB**			0.414	0.511	0.036	0.026	0.018	0.082
**SDHC**	6.29 (6.15, 6.44)	6.06 (5.91, 6.21)	0.028	0.098	0.21	0.025	0.055	0.021
**SDHD**			0.295	0.395	0.756	0.426	0.468	0.372
**SHMT1**	6.98 (6.76, 7.21)	7.32 (7.10, 7.55)	0.023	0.089	0.142	0.083	0.106	0.164
**SMOX**	6.87 (6.47, 7.27)	7.45 (7.06, 7.84)	9.50E–03	0.063	0.167	0.095	0.104	0.079
**SMS**			0.078	0.158	3.00E–03	8.40E–03	9.00E–03	2.40E–03
**SRM**			0.166	0.255	0.918	0.946	0.996	0.718
**SUCLG**	8.90 (8.77, 9.03)	9.11 (8.98, 9.24)	0.016	0.082	0.785	0.324	0.309	0.519
**TALDO1**			0.801	0.831	6.80E–03	0.017	8.80E–03	5.90E–03
**TKT**			0.086	0.158	0.149	0.083	0.117	0.059
**TPI1**	9.68 (9.43, 9.92)	9.33 (9.08, 9.57)	0.03	0.099	0.094	0.114	0.118	0.064

ACACA: Acetyl-CoA carboxylase alpha, ACO1: Aconitase 1, ACO2: Aconitase 2, ACSL1: Acyl-CoA synthetase 1, ACSL3: Acyl-CoA synthetase 3, ACSL4: Acyl-CoA synthetase 4,ACSL5: Acyl-CoA synthetase 5, ACSL6: Acyl-CoA synthetase 6, AGXT1: Alanine-glyoxylate aminotransferase 1, ALDOA: Aldolase A, ALDOB: Aldolase B, ALDOC: Aldolase C,AMD1: Adenosylmethionine decarboxylase, CHKA: Choline kinase alpha, CS: Citrate synthase, DLD: Dihydrolipoamide dehydrogenase, DLST: Dihydrolipoamides-succinyltransferase, ENO1: Enolase 1, FASN: Fatty acid synthase, FH: Fumarate hydratase, G6PD: Glucose-6-phosphate dehydrogenase, GAPDH: Glyceraldehyde-3-phosphatedehydrogenase, GPI: Glucose-6-phosphate isomerase, HK1: Hexokinase 1, HK2: Hexokinase 2, IDH1: Isocitrate dehydrogenase 1, IDH2: Isocitrate dehydrogenase 2, IDH3A:Isocitrate dehydrogenase 3A, IDH3B: Isocitrate dehydrogenase 3B, IDH3G: Isocitrate dehydrogenase 3G, MDH1: Malate dehydrogenase 1, MDH2: Malate dehydrogenase 2, NPY:Neuropeptide Y, OAZ: Ornithine decarboxylase antizyme, ODC: Ornithine decarboxylase, OGDH: Oxoglutarate (alpha-ketoglutarate) dehydrogenase, OGDHL: Oxoglutarate dehydrogenase-like, PDHA: Pyruvate dehydrogenase alpha, PDHB: Pyruvate dehydrogenase beta, PGK1: Phosphoglycerate kinase, PGD: Phosphogluconate dehydrogenase, PGLS: 6-phosphogluconolactonase, PGM1: Phosphoglucomutase, PKM2: Pyruvate kinase, RBKS: Ribokinase, RPE: Ribulose-5-phosphate-3-epimerase, RPIA: Ribose 5-phosphate isomerase, SAT1: Spermidine/spermine-N1-acetyltransferase 1, SDHA: Succinate dehydrogenase complex, subunit A, SDHB: Succinate dehydrogenase complex, subunit B, SDHD: Succinate dehydrogenase complex, subunit D, SHMT1: Serine hydroxymethyltransferase 1, SMOX: Spermine oxidase, SMS: Spermine synthase, SRM: Spermidine synthase, SUCLG: Succinyl-CoA ligase, TALDO1: Transaldolase 1, TKT: Transketolase, TPI1: Triosephosphate isomerase 1. Since the gene expression values were log2 transformed, a difference in expression by one unit corresponds to a twofold mean change in probe intensities. *Benjamini-Hochberg corrected.

Supplementary Table S8: Differences in levels of quantified metabolites in the main cohort comparing ERG_{high} samples to ERG_{low} samples in low Gleason samples (Gleason ≤ 3 + 4), corrected for multiple testing with Benjamini-Hochberg correction and adjusted for stroma, cancer, benign epithelia and luminal space

Metabolites	ERG_{low} (n = 12) Concentrations mmoles/kg wet weight, median (IQR)	ERG_{high} (n = 17) Concentrations mmoles/kg wet weight, median (IQR)	*p*-value	*p*-value*	Adjusted for stromal content	Adjusted for cancer content	Adjusted for benign epithelial content	Adjusted for luminal space content
**Alanine**	1.99 (1.64 to 2.43)	2.60 (1.78 to 2.85)	0.651	0.83	0.766	0.702	0.622	0.667
**Choline**	0.78 (0.49 to 1.43)	1.23 (0.84 to 1.48)	0.087	0.16	0.364	0.143	0.075	0.082
**Citrate**	13.95 (8.62 to 17.06)	7.07 (3.43 to 7.82)	0.001	0.004	7.70E–05	1.10E–03	9.90E–04	8.00E–04
**Creatine**	2.23 (1.63 to 2.58)	2.23 (1.68 to 2.67)	0.679	0.83	0.708	0.634	0.663	0.742
**Ethm**	0.00 (0.00 to 0.00)	0.02 (0.00 to 0.41)	0.06	0.132	0.176	0.117	0.058	0.052
**Glucose**	0.23 (0.00 to 0.43)	0.00 (0.00 to 0.07)	0.12	0.203	0.222	0.203	0.126	0.095
**Glutamate**	4.58 (3.77 to 5.48)	4.55 (4.20 to 7.79)	0.068	0.136	0.161	0.259	0.087	0.065
**Glutamine**	2.35 (2.05 to 2.68)	3.18 (2.27 to 3.66)	0.005	0.018	0.012	8.40E–03	5.20E–03	5.80E–03
**Glycine**	1.71 (1.43 to 2.22)	2.68 (1.93 to 3.51)	< 0.001	0.002	8.00E–04	4.60E–04	1.70E–04	2.90E–04
**GPC**	0.67 (0.48 to 1.18)	0.98 (0.68 to 1.24)	0.908	0.951	0.762	0.855	0.755	0.783
**GPE**	0.33 (0 to 0.53)	0.00 (0.00 to 0.73)	< 0.001	< 0.001	1.30E–40	4.10E–10	1.30E–03	6.00E–32
**Isoleucine**	0.16 (0.00 to 0.18)	0.16 (0.11 to 0.28)	0.027	0.085	5.50E–04	0.015	0.029	0.016
**Lactate**	19.77 (16.68 to 22.66)	17.81 (15.69 to 22.01)	0.997	0.997	0.845	0.833	0.984	0.984
**Leucine**	0.33 (0.26 to 0.45)	0.44 (0.28 to 0.63)	0.364	0.501	0.447	0.329	0.353	0.37
**Myo–inositol**	9.94 (7.71 to 10.25)	8.79 (7.23 to 10.09)	0.795	0.875	0.702	0.744	0.796	0.78
**PCh**	0.43 (0.26 to 0.81)	0.90 (0.54 to 1.17)	0.039	0.107	0.014	0.033	0.032	0.043
**PE**	2.18 (1.49 to 2.54)	2.63 (2.26 to 3.71)	0.055	0.132	0.016	0.053	0.039	0.034
**Putrescine**	0.20 (0.00 to 0.83)	0.05 (0.00 to 0.16)	0.001	0.004	4.60E–03	0.075	0.029	1.00E–03
**Scyllo–inositol**	0.40 (0.27 to 0.87)	0.41 (0.32 to 0.61)	0.742	0.859	0.286	0.257	0.783	0.738
**Spermine**	3.56 (1.64 to 6.07)	1.18 (0.69 to 1.54)	< 0.001	< 0.001	9.10E–08	2.00E–05	4.50E–05	5.60E–06
**Succinate**	0.51 (0.39 to 0.73)	0.62 (0.52 to 0.94)	0.151	0.237	0.325	0.191	0.142	0.133
**Taurine**	4.83 (3.20 to 6.05)	4.23 (3.03 to 4.73)	0.344	0.501	0.488	0.46	0.333	0.519
**Valine**	1.99 (1.64 to 2.43)	2.60 (1.78 to 2.85)	0.651	0.83	0.026	0.021	0.013	8.40E–03

Ethm: Ethanolamine, GPC: Glycerophosphocholine, GPE: Glycerophosphoethanolamine, PCh: Phosphocholine, PE: Phosphoethanolamine *Benjamini-Hochberg corrected.

Supplementary Table S9: Differences in levels of quantified metabolites in the main cohort comparing ERG_{high} samples to ERG_{low} samples in high Gleason samples (Gleason ≥ 4 + 3), corrected for multiple testing with Benjamini-Hochberg correction and adjusted for stroma, cancer, benign epithelia and luminal space

Metabolites	ERG_{low} (n = 18) Concentrations mmoles/kg wet weight, median (IQR)	ERG_{high} (n = 17) Concentrations mmoles/kg wet weight, median (IQR)	p–value	p value*	Adjusted for stromal content	Adjusted for cancer content	Adjusted for benign epithelial content	Adjusted for luminal space content
Alanine	2.34 (1.83 to 3.34)	2.36 (1.97 to 2.78)	0.762	0.922	0.723	0.51	0.453	0.785
Choline	1.01 (0.66 to 1.43)	1.23 (0.90 to 1.66)	0.372	0.611	0.381	0.508	0.454	0.322
Citrate	7.58 (5.51 to 10.69)	2.95 (2.34 to 4.04)	< 0.001	0.003	1.60E–04	1.00E–04	1.30E–04	3.20E–04
Creatine	2.27 (1.71 to 2.75)	1.97 (1.60 to 2.22)	0.825	0.928	0.841	0.761	0.865	0.848
Ethm	0.00 (0.00 to 0.08)	0.00 (0.00 to 0.19)	0.6	0.812	0.619	0.79	0.969	0.676
Glucose	0.15 (0.00 to 1.00)	0.00 (0.00 to 0.04)	0.016	0.087	6.10E–03	0.068	0.313	0.017
Glutamate	4.67 (4.11 to 7.28)	6.31 (5.75 to 7.74)	0.241	0.504	0.294	0.54	0.517	0.215
Glutamine	2.80 (2.45 to 3.89)	2.93 (2.69 to 3.70)	0.907	0.948	0.812	0.461	0.361	0.946
Glycine	2.57 (1.85 to 3.08)	3.17 (2.11 to 3.65)	0.366	0.611	0.389	0.58	0.833	0.417
GPC	0.81 (0.54 to 1.43)	0.84 (0.44 to 1.08)	0.714	0.912	0.798	0.886	0.908	0.38
GPE	0.00 (0.00 to 0.55)	0.00 (0.00 to 0.36)	0.541	0.778	0.538	0.518	0.507	0.413
Isoleucine	0.18 (0.07 to 0.27)	0.24 (0.12 to 0.32)	0.188	0.48	0.183	0.133	5.50E–03	0.208
Lactate	22.02 (15.64 to 27.75)	16.45 (15.70 to 20.85)	0.065	0.249	0.064	0.046	0.056	0.04
Leucine	0.52 (0.34 to 0.65)	0.59 (0.45 to 0.83)	0.974	0.974	0.978	0.86	0.828	0.966
Myo–inositol	10.82 (6.72 to 12.76)	9.39 (7.43 to 11.35)	0.226	0.504	0.236	0.226	0.401	0.377
PCh	0.72 (0.45 to 1.03)	1.12 (0.74 to 1.36)	0.019	0.087	0.021	0.079	0.232	0.011
PE	2.62 (1.35 to 3.69)	2.95 (2.78 to 3.80)	0.087	0.286	0.088	0.087	0.081	0.03
Putrescine	0.11 (0.00 to 0.56)	0.00 (0.00 to 0.00)	0.008	0.058	7.70E–03	6.80E–03	0.096	0.01
Scyllo–inositol	0.38 (0.34 to 0.59)	0.50 (0.41 to 0.60)	0.366	0.611	0.487	0.295	0.191	0.604
Spermine	1.65 (1.15 to 2.27)	0.58 (0.39 to 1.02)	< 0.001	0.004	3.10E–04	4.30E–04	3.10E–03	4.90E–04
Succinate	0.60 (0.54 to 0.74)	0.63 (0.46 to 0.76)	0.847	0.928	0.824	0.516	0.637	0.963
Taurine	6.21 (4.05 to 7.15)	4.30 (3.70 to 6.49)	0.172	0.48	0.176	0.314	0.581	0.222
Valine	0.46 (0.28 to 0.58)	0.40 (0.28 to 0.52)	0.441	0.676	0.38	0.272	0.463	0.363

Ethm: Ethanolamine, GPC: Glycerophosphocholine, GPE: Glycerophosphoethanolamine, PCh: Phosphocholine, PE: Phosphoethanolamine *Benjamini-Hochberg corrected.

Supplementary Table S10: INMEX-analysis of KEGG-pathways in the main cohort

Pathway	Total	Expected	Hits	*P*-Value	Topology
**Glutathione metabolism**	75	6.5205	17	0.000155	0.79245
**Purine metabolism**	234	20.344	33	0.00267	0.66667
**Glycerolipid metabolism**	72	6.2597	14	0.002904	0.78378
**Arginine and proline metabolism**	102	8.8679	17	0.005846	0.63441
**Cyanoamino acid metabolism**	12	1.0433	4	0.015752	1.25
**Pyruvate metabolism**	64	5.5642	11	0.019979	0.78261
**Glycolysis / Gluconeogenesis**	91	7.9116	14	0.023299	0.57627
**Glycosaminoglycan degradation**	20	1.7388	5	0.024915	0.42105
**Glycine, serine and threonine metabolism**	68	5.9119	11	0.030255	0.66667
**Pentose and glucuronate interconversions**	52	4.5209	9	0.032529	0.89655
**Lysine degradation**	73	6.3466	11	0.04783	0.46512
**beta–Alanine metabolism**	50	4.347	8	0.063613	0.38095
**Propanoate metabolism**	52	4.5209	8	0.076871	0.63415
**Glyoxylate and dicarboxylate metabolism**	53	4.6078	8	0.084079	0.52
**Valine, leucine and isoleucine degradation**	82	7.1291	11	0.094732	0.43333
**Pentose phosphate pathway**	48	4.1731	7	0.11797	0.97561
**Vitamin B6 metabolism**	15	1.3041	3	0.13563	0.4
**Ether lipid metabolism**	51	4.4339	7	0.14941	0.34483
**Fatty acid metabolism**	83	7.216	10	0.17989	1.2621
**Ascorbate and aldarate metabolism**	35	3.0429	5	0.18309	0.83333
**Alanine, aspartate and glutamate metabolism**	56	4.8686	7	0.20941	0.40816
**Aminoacyl–tRNA biosynthesis**	87	7.5638	10	0.2201	0.27536
**One carbon pool by folate**	28	2.4343	4	0.22201	0.26667
**Sphingolipid metabolism**	67	5.825	8	0.22271	0.35849
**Nicotinate and nicotinamide metabolism**	39	3.3907	5	0.24769	0.43243
**Pyrimidine metabolism**	142	12.346	15	0.24795	0.49558
**Arachidonic acid metabolism**	100	8.694	11	0.24825	0.41026
**Riboflavin metabolism**	20	1.7388	3	0.24925	0.46154
**Mucin type O–Glycan biosynthesis**	32	2.7821	4	0.30123	0.15
**Histidine metabolism**	44	3.8254	5	0.33492	0.1875
**Glycerophospholipid metabolism**	119	10.346	12	0.33741	0.37333
**Glycosphingolipid biosynthesis – ganglio series**	14	1.2172	2	0.34713	0.26087
**Glycosaminoglycan biosynthesis – chondroitin sulfate**	14	1.2172	2	0.34713	0.42857
**Pantothenate and CoA biosynthesis**	35	3.0429	4	0.3626	0.58065
**Glycosaminoglycan biosynthesis – heparan sulfate**	5	0.4347	1	0.36563	0.33333
**Butanoate metabolism**	47	4.0862	5	0.38864	0.5625
**Tryptophan metabolism**	80	6.9552	8	0.39387	0.325
**Glycosphingolipid biosynthesis – lacto and neolacto series**	26	2.2604	3	0.39675	0.067797
**Porphyrin and chlorophyll metabolism**	70	6.0858	7	0.408	0.29787
**Metabolism of xenobiotics by cytochrome P450**	139	12.085	13	0.43406	0.4
**Retinol metabolism**	83	7.216	8	0.43514	0.51163
**N–Glycan biosynthesis**	50	4.347	5	0.44211	0.17143
**Citrate cycle (TCA cycle)**	50	4.347	5	0.44211	0.75
**Butirosin and neomycin biosynthesis**	7	0.60858	1	0.47135	0.66667
**Taurine and hypotaurine metabolism**	18	1.5649	2	0.47262	0.4

**Sulfur metabolism**	19	1.6519	2	0.50172	0.5
**Drug metabolism – other enzymes**	77	6.6944	7	0.51018	0.31579
**Starch and sucrose metabolism**	78	6.7813	7	0.52435	0.35185
**Galactose metabolism**	55	4.7817	5	0.52831	0.36735
**Fructose and mannose metabolism**	55	4.7817	5	0.52831	0.43243
**Inositol phosphate metabolism**	90	7.8246	8	0.52956	0.375
**Drug metabolism – cytochrome P450**	127	11.041	11	0.55364	0.14815
**Fatty acid elongation**	57	4.9556	5	0.56115	0.35616
**Amino sugar and nucleotide sugar metabolism**	84	7.303	7	0.60576	0.2716
**Thiamine metabolism**	11	0.95634	1	0.63303	0.36364
**Valine, leucine and isoleucine biosynthesis**	13	1.1302	1	0.69432	0.72727
**Synthesis and degradation of ketone bodies**	14	1.2172	1	0.72102	0.44444
**Phenylalanine metabolism**	29	2.5213	2	0.7326	0.36364
**Caffeine metabolism**	17	1.478	1	0.78799	0.11765
**Selenocompound metabolism**	33	2.869	2	0.79592	0.21429
**Primary bile acid biosynthesis**	63	5.4772	4	0.8118	0.34043
**Glycosylphosphatidylinositol(GPI)–anchor biosynthesis**	20	1.7388	1	0.83893	0.096774
**Tyrosine metabolism**	80	6.9552	5	0.83931	0.27586
**Biosynthesis of unsaturated fatty acids**	27	2.3474	1	0.91528	0.6875
**Folate biosynthesis**	32	2.7821	1	0.94652	0.11429
**alpha–Linolenic acid metabolism**	34	2.956	1	0.95552	0.1
**Linoleic acid metabolism**	34	2.956	1	0.95552	0.25
**Terpenoid backbone biosynthesis**	39	3.3907	1	0.97196	0.057143
**Cysteine and methionine metabolism**	63	5.4772	2	0.97836	0.072727
**Fatty acid biosynthesis**	49	4.2601	1	0.98888	0.11111
**Steroid biosynthesis**	54	4.6948	1	0.99301	0.029412
**Steroid hormone biosynthesis**	137	11.911	4	0.99854	0.17978

Supplementary Table S11: INMEX-analysis of KEGG-pathways in the main cohort in low Gleason (Gleason ≤ 3 + 4) samples

Pathway	Total	Expected	Hits	P.Value	Topology
**Purine metabolism**	234	15.941	34	8.98E–06	0.57471
**Glutathione metabolism**	75	5.1091	14	0.000393	0.75472
**Pyrimidine metabolism**	142	9.6733	18	0.006584	0.58407
**Glycolysis / Gluconeogenesis**	91	6.1991	13	0.007606	0.57627
**Glycosaminoglycan degradation**	20	1.3624	5	0.009281	0.42105
**Valine, leucine and isoleucine degradation**	82	5.586	11	0.021342	0.45556
**Lysine degradation**	73	4.9729	10	0.024199	0.46512
**Arginine and proline metabolism**	102	6.9484	12	0.041654	0.31183
**Tryptophan metabolism**	80	5.4498	10	0.042465	0.375
**Cyanoamino acid metabolism**	12	0.81746	3	0.043309	1.25
**Nicotinate and nicotinamide metabolism**	39	2.6568	6	0.045681	0.48649
**beta–Alanine metabolism**	50	3.4061	7	0.050052	0.33333
**Glycerolipid metabolism**	72	4.9048	9	0.052883	0.2973
**Pentose and glucuronate interconversions**	52	3.5423	7	0.05985	0.48276
**Vitamin B6 metabolism**	15	1.0218	3	0.077143	0.4
**Sphingolipid metabolism**	67	4.5642	8	0.081965	0.32075
**Ascorbate and aldarate metabolism**	35	2.3843	5	0.08539	0.83333
**Pentose phosphate pathway**	48	3.2699	6	0.10428	0.87805
**Fatty acid metabolism**	83	5.6541	9	0.10817	1.1456
**Citrate cycle (TCA cycle)**	50	3.4061	6	0.12099	0.75
**Ether lipid metabolism**	51	3.4742	6	0.12981	0.2069
**Propanoate metabolism**	52	3.5423	6	0.13894	0.58537
**Glyoxylate and dicarboxylate metabolism**	53	3.6105	6	0.14836	0.48
**Drug metabolism – cytochrome P450**	127	8.6515	12	0.15174	0.16667
**Glycine, serine and threonine metabolism**	68	4.6323	7	0.17714	0.53968
**Amino sugar and nucleotide sugar metabolism**	84	5.7222	8	0.20982	0.39506
**Pantothenate and CoA biosynthesis**	35	2.3843	4	0.21232	0.58065
**Aminoacyl–tRNA biosynthesis**	87	5.9266	8	0.23802	0.21739
**Pyruvate metabolism**	64	4.3598	6	0.26789	0.34783
**Retinol metabolism**	83	5.6541	7	0.33519	0.37209
**Metabolism of xenobiotics by cytochrome P450**	139	9.4689	11	0.34633	0.36364
**Fatty acid elongation**	57	3.883	5	0.34699	0.35616
**Histidine metabolism**	44	2.9974	4	0.35192	0.125
**Riboflavin metabolism**	20	1.3624	2	0.39995	0.30769
**Starch and sucrose metabolism**	78	5.3135	6	0.44142	0.33333
**Arachidonic acid metabolism**	100	6.8122	7	0.5286	0.4359
**Glycosphingolipid biosynthesis – lacto and neolacto series**	26	1.7712	2	0.53782	0.050847
**Thiamine metabolism**	11	0.74934	1	0.54049	0.36364
**Biosynthesis of unsaturated fatty acids**	27	1.8393	2	0.55861	0.6875
**One carbon pool by folate**	28	1.9074	2	0.57873	0.2
**Inositol phosphate metabolism**	90	6.131	6	0.5844	0.25
**Glycosphingolipid biosynthesis – globo series**	13	0.88559	1	0.60122	0.22222
**Valine, leucine and isoleucine biosynthesis**	13	0.88559	1	0.60122	0.72727
**Drug metabolism – other enzymes**	77	5.2454	5	0.61177	0.17544

**Glycosaminoglycan biosynthesis – chondroitin sulfate**	14	0.95371	1	0.62852	0.28571
**Synthesis and degradation of ketone bodies**	14	0.95371	1	0.62852	0.44444
**Tyrosine metabolism**	80	5.4498	5	0.64615	0.22989
**N–Glycan biosynthesis**	50	3.4061	3	0.6735	0.11429
**Caffeine metabolism**	17	1.1581	1	0.69976	0.11765
**Glycerophospholipid metabolism**	119	8.1065	7	0.71401	0.17333
**Taurine and hypotaurine metabolism**	18	1.2262	1	0.72034	0.13333
**Galactose metabolism**	55	3.7467	3	0.73553	0.22449
**Fructose and mannose metabolism**	55	3.7467	3	0.73553	0.27027
**Glycosylphosphatidylinositol(GPI)–anchor biosynthesis**	20	1.3624	1	0.75739	0.096774
**Primary bile acid biosynthesis**	63	4.2917	3	0.81507	0.25532
**Butanoate metabolism**	47	3.2017	2	0.84148	0.375
**Porphyrin and chlorophyll metabolism**	70	4.7685	3	0.86718	0.10638
**Phenylalanine metabolism**	29	1.9755	1	0.8722	0.18182
**Mucin type O–Glycan biosynthesis**	32	2.1799	1	0.89684	0.15
**Alanine, aspartate and glutamate metabolism**	56	3.8148	2	0.90443	0.12245
**Linoleic acid metabolism**	34	2.3161	1	0.91058	0.5
**Cysteine and methionine metabolism**	63	4.2917	2	0.93638	0.10909
**Steroid hormone biosynthesis**	137	9.3327	2	0.99944	0.10112

Supplementary Table S12: INMEX-analysis of KEGG-pathways in the main cohort in high Gleason (Gleason ≥ 4 + 3) samples

Pathway	Total	Expected	Hits	*P*-Value	Topology
**Purine metabolism**	234	5.4603	13	0.002268	0.41954
**Pyrimidine metabolism**	142	3.3135	9	0.004933	0.49558
**Glutathione metabolism**	75	1.7501	6	0.007251	0.49057
**Glycosphingolipid biosynthesis – lacto and neolacto series**	26	0.6067	3	0.02147	0.067797
**Nicotinate and nicotinamide metabolism**	39	0.91005	3	0.061006	0.37838
**Glycine, serine and threonine metabolism**	68	1.5868	4	0.072199	0.2381
**Arginine and proline metabolism**	102	2.3801	5	0.087048	0.16129
**Retinol metabolism**	83	1.9368	4	0.1264	0.37209
**Arachidonic acid metabolism**	100	2.3335	4	0.20307	0.12821
**Cyanoamino acid metabolism**	12	0.28002	1	0.24718	0.5
**Drug metabolism – other enzymes**	77	1.7968	3	0.26692	0.10526
**Glycosaminoglycan biosynthesis – chondroitin sulfate**	14	0.32668	1	0.28207	0.14286
**Glycosphingolipid biosynthesis – ganglio series**	14	0.32668	1	0.28207	0.17391
**Vitamin B6 metabolism**	15	0.35002	1	0.29891	0.1
**Fatty acid metabolism**	83	1.9368	3	0.3056	0.19417
**N–Glycan biosynthesis**	50	1.1667	2	0.32651	0.057143
**Citrate cycle (TCA cycle)**	50	1.1667	2	0.32651	0.29545
**Caffeine metabolism**	17	0.39669	1	0.33143	0.11765
**Drug metabolism – cytochrome P450**	127	2.9635	4	0.3441	0.50926
**Taurine and hypotaurine metabolism**	18	0.42002	1	0.34713	0.13333
**Inositol phosphate metabolism**	90	2.1001	3	0.35105	0.125
**Glyoxylate and dicarboxylate metabolism**	53	1.2367	2	0.35238	0.12
**Fructose and mannose metabolism**	55	1.2834	2	0.36944	0.16216
**Riboflavin metabolism**	20	0.46669	1	0.37746	0.15385
**Metabolism of xenobiotics by cytochrome P450**	139	3.2435	4	0.40908	0.36364
**One carbon pool by folate**	28	0.65337	1	0.48549	0.2
**Glycerolipid metabolism**	72	1.6801	2	0.50592	0.18919
**Lysine degradation**	73	1.7034	2	0.51337	0.13953
**Folate biosynthesis**	32	0.74671	1	0.53236	0.11429
**Mucin type O–Glycan biosynthesis**	32	0.74671	1	0.53236	0.15
**Linoleic acid metabolism**	34	0.79338	1	0.55419	0.5
**Tryptophan metabolism**	80	1.8668	2	0.56346	0.05
**Tyrosine metabolism**	80	1.8668	2	0.56346	0.068966
**Pantothenate and CoA biosynthesis**	35	0.81671	1	0.56473	0.12903
**Valine, leucine and isoleucine degradation**	82	1.9134	2	0.57709	0.044444
**beta–Alanine metabolism**	50	1.1667	1	0.6963	0.047619
**Ether lipid metabolism**	51	1.1901	1	0.70352	0.068966
**Pentose and glucuronate interconversions**	52	1.2134	1	0.71058	0.13793
**Alanine, aspartate and glutamate metabolism**	56	1.3067	1	0.73718	0.081633
**Fatty acid elongation**	57	1.3301	1	0.74344	0.041096
**Primary bile acid biosynthesis**	63	1.4701	1	0.77806	0.19149
**Sphingolipid metabolism**	67	1.5634	1	0.79854	0.037736
**Porphyrin and chlorophyll metabolism**	70	1.6334	1	0.81267	0.042553
**Steroid hormone biosynthesis**	137	3.1968	2	0.83921	0.05618

**Starch and sucrose metabolism**	78	1.8201	1	0.84576	0.037037
**Amino sugar and nucleotide sugar metabolism**	84	1.9601	1	0.86673	0.049383
**Glycolysis / Gluconeogenesis**	91	2.1234	1	0.88767	0.067797
**Glycerophospholipid metabolism**	119	2.7768	1	0.94357	0.053333

Supplementary Table S13: GSEA of KEGG-pathways in the main cohort, enrichment in ERG_{low}

PATHWAY	SIZE	ES	NES	NOM p-val	FDR *q*-val
**DRUG METABOLISM CYTOCHROME P450**	55	–0.6651254	–1.7795105	0.002132196	0.38855085
**GLYCOLYSIS GLUCONEOGENESIS**	56	–0.4682116	–1.6126508	0.013100437	0.8536236
**HEMATOPOIETIC CELL LINEAGE**	68	–0.6377796	–1.5941937	0.01632653	0.66214764
**BETA ALANINE METABOLISM**	19	–0.6408701	–1.563926	0.020242915	0.62843364
**GLUTATHIONE METABOLISM**	45	–0.5840763	–1.5582185	0.052738335	0.528509
**PRION DISEASES**	30	–0.5652002	–1.5239826	0.03285421	0.57103133
**HYPERTROPHIC CARDIOMYOPATHY HCM**	66	–0.5525483	–1.521536	0.04592902	0.49907997
**CARDIAC MUSCLE CONTRACTION**	58	–0.5234164	–1.5056521	0.033126295	0.4885571
**METABOLISM OF XENOBIOTICS BY CYTOCHROME P450**	50	–0.5358754	–1.4941844	0.017094018	0.47057903
**VALINE LEUCINE AND ISOLEUCINE DEGRADATION**	44	–0.556476	–1.491827	0.0741483	0.43163717
**PPAR SIGNALING PATHWAY**	51	–0.52109236	–1.4773304	0.027139874	0.43209052
**FATTY ACID METABOLISM**	35	–0.4665317	–1.4668512	0.07385229	0.42450482
**DILATED CARDIOMYOPATHY**	73	–0.51889294	–1.4528376	0.07660455	0.4315167
**HISTIDINE METABOLISM**	24	–0.5824359	–1.4319005	0.043912176	0.4642199
**APOPTOSIS**	75	–0.35446638	–1.4161711	0.0503876	0.48000923
**TYROSINE METABOLISM**	31	–0.51137507	–1.3706481	0.07236842	0.5867568
**RETINOL METABOLISM**	40	–0.4578147	–1.3549248	0.06313646	0.6055808
**ARGININE AND PROLINE METABOLISM**	46	–0.47907144	–1.3485328	0.09325397	0.5914392
**PROXIMAL TUBULE BICARBONATE RECLAMATION**	18	–0.5100017	–1.3183355	0.11677282	0.6578999
**ALDOSTERONE REGULATED SODIUM REABSORPTION**	33	–0.5453702	–1.3126537	0.1122449	0.64245903
**GLYCOSPHINGOLIPID BIOSYNTHESIS LACTO AND NEOLACTO SERIES**	17	–0.47345862	–1.2855277	0.15303983	0.6999029
**PROPANOATE METABOLISM**	31	–0.42541942	–1.2627256	0.2413793	0.74142903
**MELANOGENESIS**	82	–0.38168576	–1.2602468	0.12931034	0.71696585
**COLORECTAL CANCER**	54	–0.3426833	–1.2553287	0.16260162	0.7035733
**NICOTINATE AND NICOTINAMIDE METABOLISM**	19	–0.5683716	–1.249494	0.15118791	0.69425493
**REGULATION OF ACTIN CYTOSKELETON**	176	–0.32957068	–1.2489338	0.18371607	0.6691262
**MTOR SIGNALING PATHWAY**	43	–0.32117867	–1.2314632	0.15748031	0.7004713
**FOCAL ADHESION**	168	–0.38118944	–1.2233069	0.20910972	0.70139503
**STEROID HORMONE BIOSYNTHESIS**	36	–0.44694844	–1.2228109	0.16115703	0.6786272
**B CELL RECEPTOR SIGNALING PATHWAY**	70	–0.39102882	–1.2056568	0.24158415	0.7087562
**GLIOMA**	57	–0.33602458	–1.2041162	0.16297787	0.6909752
**PANCREATIC CANCER**	63	–0.33796537	–1.2031119	0.21850394	0.67281705
**PHENYLALANINE METABOLISM**	17	–0.50094825	–1.1898077	0.21638656	0.6888747
**CELL ADHESION MOLECULES CAMS**	112	–0.4456652	–1.1702002	0.26814517	0.7239068

**VASCULAR SMOOTH MUSCLE CONTRACTION**	89	–0.3525924	–1.1684161	0.2516269	0.70892125
**PHOSPHATIDYLINOSITOL SIGNALING SYSTEM**	65	–0.32815862	–1.1536041	0.26326963	0.73398
**NATURAL KILLER CELL MEDIATED CYTOTOXICITY**	101	–0.3900806	–1.1503791	0.3156823	0.72411835
**MAPK SIGNALING PATHWAY**	218	–0.2851731	–1.1377566	0.22937626	0.74028134
**ADHERENS JUNCTION**	62	–0.31532508	–1.1332755	0.2838983	0.7337195
**WNT SIGNALING PATHWAY**	129	–0.31501043	–1.1323106	0.24347825	0.7179757
**ANTIGEN PROCESSING AND PRESENTATION**	65	–0.43509716	–1.1301155	0.33467743	0.7065309
**TRYPTOPHAN METABOLISM**	33	–0.43367508	–1.1286054	0.27272728	0.6938948
**PYRUVATE METABOLISM**	38	–0.31179887	–1.1273845	0.3224401	0.68096334
**CHEMOKINE SIGNALING PATHWAY**	152	–0.3680516	–1.1199098	0.31451613	0.683802
**ARRHYTHMOGENIC RIGHT VENTRICULAR CARDIOMYOPATHY ARVC**	59	–0.38263643	–1.1110188	0.32415253	0.6903247
**PROSTATE CANCER**	83	–0.2898831	–1.1048385	0.30181086	0.69068277
**P53 SIGNALING PATHWAY**	61	–0.36322063	–1.1022831	0.31013918	0.6820439
**VIRAL MYOCARDITIS**	62	–0.4290487	–1.102143	0.35918367	0.66820705
**CALCIUM SIGNALING PATHWAY**	124	–0.32479998	–1.0972714	0.29817444	0.666126
**LEISHMANIA INFECTION**	58	–0.41226605	–1.094746	0.3478261	0.6588297
**PYRIMIDINE METABOLISM**	90	–0.27208874	–1.0883919	0.33539096	0.66017336
**ENDOCYTOSIS**	156	–0.25955555	–1.080931	0.3253012	0.6651845
**FC EPSILON RI SIGNALING PATHWAY**	62	–0.33219257	–1.0740509	0.35458168	0.66915274
**INOSITOL PHOSPHATE METABOLISM**	49	–0.29495132	–1.0660963	0.36134455	0.6755806
**SNARE INTERACTIONS IN VESICULAR TRANSPORT**	34	–0.33910057	–1.059901	0.40361446	0.6771761
**LEUKOCYTE TRANSENDOTHELIAL MIGRATION**	94	–0.3551334	–1.0428008	0.3970894	0.70556414
**PARKINSONS DISEASE**	103	–0.23767659	–1.0376086	0.39478958	0.70493156
**ASCORBATE AND ALDARATE METABOLISM**	18	–0.4042498	–1.0335944	0.41910332	0.7018473
**FC GAMMA R MEDIATED PHAGOCYTOSIS**	83	–0.29817593	–1.0322497	0.39959016	0.69348305
**TIGHT JUNCTION**	106	–0.27402833	–1.0200877	0.41908714	0.7086325
**PATHWAYS IN CANCER**	279	–0.26002625	–1.0173993	0.40248963	0.7030485
**HEDGEHOG SIGNALING PATHWAY**	39	–0.38406587	–1.0157797	0.40900195	0.69598156
**OXIDATIVE PHOSPHORYLATION**	107	–0.22497028	–1.0146433	0.42720306	0.68765503
**PORPHYRIN AND CHLOROPHYLL METABOLISM**	33	–0.33246934	–1.0108454	0.4486166	0.6846646
**NEUROTROPHIN SIGNALING PATHWAY**	114	–0.224331	–1.007843	0.4305835	0.6805984
**EPITHELIAL CELL SIGNALING IN HELICOBACTER PYLORI INFECTION**	65	–0.25337642	–0.9983357	0.4385246	0.69139266
**COMPLEMENT AND COAGULATION CASCADES**	52	–0.4048311	–0.9914725	0.46666667	0.69601685
**RENAL CELL CARCINOMA**	61	–0.27857324	–0.98580015	0.47572815	0.69756716
**LINOLEIC ACID METABOLISM**	16	–0.4168835	–0.97966015	0.47358122	0.7009005
**GRAFT VERSUS HOST DISEASE**	31	–0.47203773	–0.97773933	0.47912526	0.6949405
**LONG TERM POTENTIATION**	60	–0.29623806	–0.97161144	0.49275362	0.69866616
**MELANOMA**	58	–0.26884857	–0.9689658	0.52165353	0.6946373
**PRIMARY IMMUNODEFICIENCY**	29	–0.46399686	–0.9642447	0.5423387	0.6954509
**ARACHIDONIC ACID METABOLISM**	40	–0.3602123	–0.94764656	0.5665962	0.72030175
**AXON GUIDANCE**	119	–0.26357418	–0.9413953	0.55737704	0.723444
**HUNTINGTONS DISEASE**	157	–0.20142505	–0.9413445	0.53816044	0.7140554
**CYTOKINE CYTOKINE RECEPTOR INTERACTION**	183	–0.31934986	–0.93090165	0.5535714	0.7261811

**ALLOGRAFT REJECTION**	26	–0.48169452	–0.92606074	0.56363636	0.7267475
**BASAL CELL CARCINOMA**	40	–0.32223517	–0.9183921	0.59163344	0.733288
**NOD LIKE RECEPTOR SIGNALING PATHWAY**	48	–0.3053208	–0.9062703	0.5923077	0.7488158
**DRUG METABOLISM OTHER ENZYMES**	39	–0.30631727	–0.898475	0.6105675	0.755737
**T CELL RECEPTOR SIGNALING PATHWAY**	92	–0.26117206	–0.88521665	0.58171207	0.77357084
**BUTANOATE METABOLISM**	30	–0.35817483	–0.882406	0.62896824	0.76976794
**TERPENOID BACKBONE BIOSYNTHESIS**	15	–0.365085	–0.8818788	0.6188605	0.76160514
**SYSTEMIC LUPUS ERYTHEMATOSUS**	88	–0.31776744	–0.88100976	0.6051081	0.7542704
**LYSOSOME**	117	–0.23417728	–0.87776816	0.6113281	0.7519492
**ECM RECEPTOR INTERACTION**	70	–0.33946648	–0.86563253	0.64656967	0.7670777
**RNA POLYMERASE**	27	–0.26543185	–0.86558276	0.63723606	0.75846314
**TYPE I DIABETES MELLITUS**	32	–0.40558252	–0.8645699	0.6372549	0.75184464
**TOLL LIKE RECEPTOR SIGNALING PATHWAY**	76	–0.2723002	–0.86427546	0.63527054	0.74425393
**AUTOIMMUNE THYROID DISEASE**	34	–0.40742585	–0.8585488	0.6356275	0.7463378
**JAK STAT SIGNALING PATHWAY**	114	–0.24149683	–0.84474975	0.6859504	0.7643172
**GALACTOSE METABOLISM**	20	–0.24985434	–0.8222984	0.67701864	0.79740804
**DORSO VENTRAL AXIS FORMATION**	23	–0.27150398	–0.81813145	0.68136275	0.7966562
**ASTHMA**	21	–0.38351947	–0.7867748	0.7321063	0.8421756
**PEROXISOME**	67	–0.2196404	–0.7829821	0.7924528	0.8402849
**INTESTINAL IMMUNE NETWORK FOR IGA PRODUCTION**	34	–0.34740373	–0.7700342	0.7526427	0.85196656
**PATHOGENIC ESCHERICHIA COLI INFECTION**	50	–0.2641702	–0.7688507	0.7628866	0.8450977
**O GLYCAN BIOSYNTHESIS**	19	–0.28100225	–0.75185394	0.8367347	0.8638152
**VIBRIO CHOLERAE INFECTION**	51	–0.19520424	–0.7430977	0.8526971	0.868085
**TASTE TRANSDUCTION**	25	–0.2806909	–0.72318774	0.8623482	0.88615614
**STEROID BIOSYNTHESIS**	16	–0.2878557	–0.7064701	0.83657587	0.8987915
**CYTOSOLIC DNA SENSING PATHWAY**	42	–0.2202738	–0.6797214	0.86875	0.92031956
**CELL CYCLE**	108	–0.17019337	–0.63154733	0.9644269	0.9548837
**GLYCOSYLPHOSPHATIDYLINOSITOL GPI ANCHOR BIOSYNTHESIS**	24	–0.20134689	–0.5982353	0.868	0.9676463
**PROTEASOME**	41	–0.16021891	–0.54751354	0.8828125	0.97920084

Supplementary Table S14: GSEA of KEGG-pathways in the main cohort, enrichment in ERG_{high}

PATHWAY	SIZE	ES	NES	NOM *p*-val	FDR *q*-val
NOTCH SIGNALING PATHWAY	44	0.570646	1.8939956	0	0.055041593
PENTOSE AND GLUCURONATE INTERCONVERSIONS	22	0.539804	1.477712	0.07157895	1
CITRATE CYCLE TCA CYCLE	31	0.379956	1.4168941	0.097087376	1
SPLICEOSOME	106	0.315289	1.4022022	0.15665236	1
ENDOMETRIAL CANCER	45	0.37221	1.3950136	0.084337346	0.9674002
GLYCOSAMINOGLYCAN DEGRADATION	19	0.442775	1.3926028	0.120527305	0.8168873
RNA DEGRADATION	52	0.31642	1.3648297	0.15400411	0.82250255
TYPE II DIABETES MELLITUS	32	0.417495	1.3610706	0.057692308	0.7334166
ETHER LIPID METABOLISM	23	0.463213	1.2851268	0.14164905	0.97442824
SPHINGOLIPID METABOLISM	33	0.407494	1.283116	0.092929296	0.88598776
N GLYCAN BIOSYNTHESIS	43	0.387991	1.2674928	0.21501014	0.86686134
ADIPOCYTOKINE SIGNALING PATHWAY	56	0.352025	1.2573285	0.11890838	0.83355254
ABC TRANSPORTERS	35	0.405498	1.2435976	0.17958412	0.81864834
SELENOAMINO ACID METABOLISM	21	0.420925	1.2259183	0.25940594	0.82157844
VEGF SIGNALING PATHWAY	61	0.344259	1.2232822	0.16765286	0.77542835
GLYCOSAMINOGLYCAN BIOSYNTHESIS HEPARAN SULFATE	20	0.382288	1.219865	0.19569471	0.73947895
NITROGEN METABOLISM	16	0.533677	1.219607	0.1902834	0.6969087
PURINE METABOLISM	137	0.295115	1.2109817	0.15090543	0.68312067
GLYCEROLIPID METABOLISM	38	0.354304	1.1863607	0.17751479	0.720392
BASE EXCISION REPAIR	32	0.315088	1.1661458	0.24395162	0.7446007
PROGESTERONE MEDIATED OOCYTE MATURATION	74	0.331903	1.1658318	0.248	0.7097456
AMINOACYL TRNA BIOSYNTHESIS	40	0.340565	1.1531861	0.3215859	0.7137402
LYSINE DEGRADATION	41	0.305711	1.136898	0.27131784	0.72547805
ERBB SIGNALING PATHWAY	74	0.268035	1.1329427	0.23326571	0.7061263
LONG TERM DEPRESSION	53	0.325049	1.1232173	0.26185566	0.70176786
CHRONIC MYELOID LEUKEMIA	65	0.289681	1.1228493	0.29545453	0.6758072
GAP JUNCTION	73	0.349332	1.1220355	0.29400387	0.6528848
GLYCOSAMINOGLYCAN BIOSYNTHESIS CHONDROITIN SULFATE	19	0.43838	1.1136811	0.30812854	0.6488753
UBIQUITIN MEDIATED PROTEOLYSIS	127	0.247331	1.1013335	0.3417191	0.6578307
INSULIN SIGNALING PATHWAY	114	0.258285	1.1009762	0.3029703	0.63664
RIBOSOME	82	0.305605	1.0945854	0.37787056	0.63081235
PROTEIN EXPORT	22	0.379509	1.0859424	0.39918533	0.6306105
VASOPRESSIN REGULATED WATE R REABSORPTION	38	0.288508	1.0723445	0.3472222	0.64245826
GLYOXYLATE AND DICARBOXYLATE METABOLISM	15	0.368145	1.0647148	0.4090909	0.63977957
BLADDER CANCER	41	0.338338	1.0609249	0.3580786	0.62997067
GLYCEROPHOSPHOLIPID METABOLISM	59	0.260367	1.0561461	0.36507937	0.622571
PENTOSE PHOSPHATE PATHWAY	24	0.308604	1.024085	0.44421053	0.6753275
ACUTE MYELOID LEUKEMIA	53	0.268619	1.0056236	0.43217054	0.6977903
RIG I LIKE RECEPTOR SIGNALING PATHWAY	54	0.284634	1.003308	0.44399184	0.6847781

**GLYCINE SERINE AND THREONINE METABOLISM**	29	0.363609	0.99739045	0.4529058	0.67987096
**NEUROACTIVE LIGAND RECEPTOR INTERACTION**	144	0.290831	0.9860564	0.4940476	0.6868037
**MISMATCH REPAIR**	22	0.296132	0.97391146	0.47268906	0.69548035
**STARCH AND SUCROSE METABOLISM**	38	0.297813	0.96517473	0.53195876	0.6982206
**GNRH SIGNALING PATHWAY**	84	0.261413	0.95846295	0.53154874	0.69538563
**THYROID CANCER**	29	0.288496	0.9438243	0.5331992	0.7112361
**OOCYTE MEIOSIS**	98	0.252336	0.94085276	0.5365854	0.7022131
**BASAL TRANSCRIPTION FACTORS**	28	0.243772	0.91830355	0.5484536	0.7325656
**ONE CARBON POOL BY FOLATE**	16	0.356132	0.9163467	0.552521	0.7210434
**NON SMALL CELL LUNG CANCER**	50	0.249019	0.9008779	0.5807087	0.7362479
**TGF BETA SIGNALING PATHWAY**	71	0.297991	0.88788795	0.6529081	0.74723715
**SMALL CELL LUNG CANCER**	77	0.248611	0.87011284	0.6429942	0.7668747
**REGULATION OF AUTOPHAGY**	24	0.226453	0.8181001	0.70623744	0.85259765
**AMINO SUGAR AND NUCLEOTIDE SUGAR METABOLISM**	41	0.236854	0.80509555	0.7211155	0.8610756
**ALZHEIMERS DISEASE**	141	0.201338	0.8043591	0.69246435	0.8465044
**AMYOTROPHIC LATERAL SCLEROSIS ALS**	44	0.249455	0.75974053	0.82077396	0.9104104
**ALANINE ASPARTATE AND GLUTAMATE METABOLISM**	28	0.253045	0.73419213	0.8477801	0.93348867
**CYSTEINE AND METHIONINE METABOLISM**	29	0.212136	0.72075784	0.8646465	0.9367848
**HOMOLOGOUS RECOMBINATION**	24	0.218325	0.7071195	0.8479657	0.9396755
**FRUCTOSE AND MANNOSE METABOLISM**	30	0.222205	0.70003974	0.88957053	0.93191516
**BIOSYNTHESIS OF UNSATURATED FATTY ACIDS**	20	0.278155	0.6484301	0.8965517	0.97063565
**NUCLEOTIDE EXCISION REPAIR**	42	0.1579	0.6299698	0.9227557	0.97013724
**DNA REPLICATION**	36	0.18302	0.5928331	0.9354839	0.9776261
**OLFACTORY TRANSDUCTION**	131	0.168927	0.4935225	0.989899	0.99249303

Supplementary Table S15: GSEA of KEGG-pathways in the main cohort, low Gleason (Gleason ≤ 3 + 4) samples, enrichment in ERG_{low}

PATHWAY	SIZE	ES	NES	NOM *p*-val	FDR *q*-val
FATTY ACID METABOLISM	35	–0.5748	–1.75733	0.003976	0.428658
VALINE LEUCINE AND ISOLEUCINE DEGRADATION	44	–0.67001	–1.73347	0.006342	0.270427
GLYCOLYSIS GLUCONEOGENESIS	56	–0.53319	–1.7318	0.00396	0.183222
DRUG METABOLISM CYTOCHROME P450	55	–0.64306	–1.6711	0.002024	0.243507
PROPANOATE METABOLISM	31	–0.53258	–1.66536	0.037815	0.209191
HEMATOPOIETIC CELL LINEAGE	68	–0.65124	–1.63053	0	0.240902
PPAR SIGNALING PATHWAY	51	–0.56741	–1.58869	0.002151	0.294395
TYROSINE METABOLISM	31	–0.55823	–1.51035	0.022587	0.467732
HISTIDINE METABOLISM	24	–0.62194	–1.50656	0.026639	0.430377
GLUTATHIONE METABOLISM	45	–0.55473	–1.49415	0.056863	0.42461
BETA ALANINE METABOLISM	19	–0.62699	–1.49271	0.056604	0.389144
METABOLISM OF XENOBIOTICS BY CYTOCHROME P450	50	–0.53636	–1.48458	0.02988	0.377124
RETINOL METABOLISM	40	–0.52352	–1.47665	0.02268	0.369106
CARDIAC MUSCLE CONTRACTION	58	–0.54267	–1.4481	0.076152	0.417485
HYPERTROPHIC CARDIOMYOPATHY HCM	66	–0.54303	–1.43106	0.057613	0.436884
PYRUVATE METABOLISM	38	–0.3643	–1.41729	0.085774	0.44844
DILATED CARDIOMYOPATHY	73	–0.52784	–1.39136	0.08125	0.498288
NICOTINATE AND NICOTINAMIDE METABOLISM	19	–0.63499	–1.37941	0.079399	0.50922
ARACHIDONIC ACID METABOLISM	40	–0.49731	–1.36389	0.025105	0.526413
VIRAL MYOCARDITIS	62	–0.52378	–1.36242	0.130952	0.50457
CELL ADHESION MOLECULES CAMS	112	–0.51387	–1.34666	0.116564	0.523981
B CELL RECEPTOR SIGNALING PATHWAY	70	–0.41894	–1.3323	0.165975	0.54144
BUTANOATE METABOLISM	30	–0.50098	–1.32435	0.136821	0.540635
PRIMARY IMMUNODEFICIENCY	29	–0.59903	–1.31789	0.184874	0.535373
PROXIMAL TUBULE BICARBONATE RECLAMATION	18	–0.4973	–1.30566	0.121339	0.549594
FC EPSILON RI SIGNALING PATHWAY	62	–0.39485	–1.29086	0.122699	0.572681
VASCULAR SMOOTH MUSCLE CONTRACTION	89	–0.39253	–1.2908	0.164905	0.551471
REGULATION OF ACTIN CYTOSKELETON	176	–0.34923	–1.28702	0.145791	0.541866
GLYCOSPHINGOLIPID BIOSYNTHESIS LACTO AND NEOLACTO SERIES	17	–0.49558	–1.28193	0.158635	0.535908
LEISHMANIA INFECTION	58	–0.48024	–1.26	0.188017	0.579365
OXIDATIVE PHOSPHORYLATION	107	–0.25188	–1.25928	0.223158	0.563622
LINOLEIC ACID METABOLISM	16	–0.52279	–1.25469	0.152361	0.556829
PHENYLALANINE METABOLISM	17	–0.5513	–1.25461	0.163223	0.540131
LEUKOCYTE TRANSENDOTHELIAL MIGRATION	94	–0.42807	–1.25319	0.160825	0.527582
ADHERENS JUNCTION	62	–0.35074	–1.24188	0.177291	0.539059
NATURAL KILLER CELL MEDIATED CYTOTOXICITY	101	–0.40518	–1.24066	0.214592	0.527223
EPITHELIAL CELL SIGNALING IN HELICOBACTER PYLORI INFECTION	65	–0.28888	–1.24058	0.131466	0.5131
PARKINSONS DISEASE	103	–0.26831	–1.24027	0.235294	0.500656
FC GAMMA R MEDIATED PHAGOCYTOSIS	83	–0.34748	–1.22995	0.207039	0.512762
CHEMOKINE SIGNALING PATHWAY	152	–0.39003	–1.22606	0.202479	0.509208
FOCAL ADHESION	168	–0.37517	–1.22213	0.194861	0.505357
PHOSPHATIDYLINOSITOL SIGNALING SYSTEM	65	–0.34458	–1.2177	0.169661	0.50295

**PANCREATIC CANCER**	63	–0.34855	–1.21172	0.208511	0.505366
**ENDOCYTOSIS**	156	–0.28012	–1.20086	0.170782	0.517217
**ANTIGEN PROCESSING AND PRESENTATION**	65	–0.46206	–1.19376	0.287234	0.522465
**PYRIMIDINE METABOLISM**	90	–0.2891	–1.18693	0.221766	0.525949
**ARGININE AND PROLINE METABOLISM**	46	–0.40073	–1.18367	0.189655	0.522112
**PRION DISEASES**	30	–0.43579	–1.18351	0.240816	0.511485
**ASCORBATE AND ALDARATE METABOLISM**	18	–0.47857	–1.18189	0.273092	0.504342
**SYSTEMIC LUPUS ERYTHEMATOSUS**	88	–0.41173	–1.17882	0.237323	0.500253
**STEROID HORMONE BIOSYNTHESIS**	36	–0.43597	–1.17244	0.224	0.503355
**WNT SIGNALING PATHWAY**	129	–0.31684	–1.1669	0.225873	0.505087
**TIGHT JUNCTION**	106	–0.32125	–1.14441	0.243083	0.543737
**MTOR SIGNALING PATHWAY**	43	–0.29399	–1.14222	0.273101	0.537842
**MELANOGENESIS**	82	–0.34923	–1.13872	0.264151	0.535669
**GLYCEROLIPID METABOLISM**	38	–0.33329	–1.12194	0.278481	0.561586
**LYSOSOME**	117	–0.28709	–1.1122	0.317328	0.571848
**MAPK SIGNALING PATHWAY**	218	–0.28331	–1.10765	0.269737	0.571201
**ALDOSTERONE REGULATED SODIUM REABSORPTION**	33	–0.45758	–1.10048	0.335541	0.576414
**INOSITOL PHOSPHATE METABOLISM**	49	–0.31283	–1.09895	0.331959	0.569941
**TOLL LIKE RECEPTOR SIGNALING PATHWAY**	76	–0.32024	–1.09699	0.316222	0.565621
**COLORECTAL CANCER**	54	–0.29807	–1.08723	0.327766	0.575742
**SNARE INTERACTIONS IN VESICULAR TRANSPORT**	34	–0.3634	–1.07756	0.382892	0.586732
**TYPE I DIABETES MELLITUS**	32	–0.52346	–1.07534	0.40257	0.582921
**APOPTOSIS**	75	–0.26781	–1.06279	0.335484	0.600432
**VIBRIO CHOLERAE INFECTION**	51	–0.26823	–1.06249	0.331276	0.591874
**CYTOKINE CYTOKINE RECEPTOR INTERACTION**	183	–0.35607	–1.05478	0.377682	0.598656
**TRYPTOPHAN METABOLISM**	33	–0.40505	–1.05363	0.368973	0.592153
**CALCIUM SIGNALING PATHWAY**	124	–0.31068	–1.05021	0.368644	0.590896
**HEDGEHOG SIGNALING PATHWAY**	39	–0.38985	–1.04884	0.378099	0.584801
**T CELL RECEPTOR SIGNALING PATHWAY**	92	–0.28932	–1.04161	0.397872	0.591842
**AUTOIMMUNE THYROID DISEASE**	34	–0.49558	–1.04001	0.440426	0.586462
**ASTHMA**	21	–0.50737	–1.03382	0.428571	0.59034
**ALLOGRAFT REJECTION**	26	–0.54805	–1.0317	0.471215	0.586535
**AXON GUIDANCE**	119	–0.27966	–1.03134	0.36646	0.579415
**JAK STAT SIGNALING PATHWAY**	114	–0.28618	–1.02503	0.417671	0.584776
**ARRHYTHMOGENIC RIGHT VENTRICULAR CARDIOMYOPATHY ARVC**	59	–0.34725	–1.02209	0.430353	0.582792
**GLIOMA**	57	–0.28234	–1.02111	0.436059	0.57718
**COMPLEMENT AND COAGULATION CASCADES**	52	–0.42698	–1.02096	0.448637	0.570092
**STARCH AND SUCROSE METABOLISM**	38	–0.316	–1.02012	0.415612	0.564567
**GRAFT VERSUS HOST DISEASE**	31	–0.50635	–1.01979	0.487288	0.558214
**GAP JUNCTION**	73	–0.30982	–1.01693	0.409871	0.556332
**ADIPOCYTOKINE SIGNALING PATHWAY**	56	–0.27146	–1.01226	0.435374	0.558633
**PATHWAYS IN CANCER**	279	–0.25379	–1.00408	0.418605	0.567434
**LONG TERM DEPRESSION**	53	–0.29765	–0.99491	0.431535	0.577069
**NON SMALL CELL LUNG CANCER**	50	–0.27431	–0.99275	0.494759	0.574468
**PATHOGENIC ESCHERICHIA COLI INFECTION**	50	–0.32015	–0.9716	0.483607	0.606761
**GNRH SIGNALING PATHWAY**	84	–0.26309	–0.96915	0.493802	0.605055

MELANOMA	58	–0.27091	–0.95161	0.561181	0.630692
LONG TERM POTENTIATION	60	–0.27998	–0.9468	0.544699	0.632693
FRUCTOSE AND MANNOSE METABOLISM	30	–0.29582	–0.94543	0.543651	0.628576
GLYCEROPHOSPHOLIPID METABOLISM	59	–0.23849	–0.93851	0.57551	0.635078
HUNTINGTONS DISEASE	157	–0.19111	–0.93586	0.507592	0.633028
RENAL CELL CARCINOMA	61	–0.25307	–0.93248	0.541667	0.632276
BASAL CELL CARCINOMA	40	–0.31747	–0.92139	0.60334	0.64651
ALZHEIMERS DISEASE	141	–0.22165	–0.9201	0.53139	0.642262
VEGF SIGNALING PATHWAY	61	–0.25292	–0.91574	0.622129	0.643416
TERPENOID BACKBONE BIOSYNTHESIS	15	–0.36513	–0.90732	0.578189	0.650887
PROSTATE CANCER	83	–0.24162	–0.90552	0.597895	0.647442
PORPHYRIN AND CHLOROPHYLL METABOLISM	33	–0.30215	–0.89355	0.612774	0.662094
PEROXISOME	67	–0.24244	–0.88397	0.659919	0.67278
AMINO SUGAR AND NUCLEOTIDE SUGAR METABOLISM	41	–0.24287	–0.88212	0.675676	0.669515
GALACTOSE METABOLISM	20	–0.2704	–0.87744	0.612971	0.671198
DRUG METABOLISM OTHER ENZYMES	39	–0.30971	–0.87682	0.638211	0.665909
INTESTINAL IMMUNE NETWORK FOR IGA PRODUCTION	34	–0.3731	–0.84708	0.645435	0.711469
STEROID BIOSYNTHESIS	16	–0.34029	–0.81211	0.696121	0.764039
RNA POLYMERASE	27	–0.23329	–0.79392	0.72211	0.787158
P53 SIGNALING PATHWAY	61	–0.25305	–0.78393	0.795031	0.796277
ALANINE ASPARTATE AND GLUTAMATE METABOLISM	28	–0.26272	–0.78148	0.796334	0.792567
BIOSYNTHESIS OF UNSATURATED FATTY ACIDS	20	–0.30046	–0.72265	0.814196	0.869183
GLYCOSYLPHOSPHATIDYLINOSITOL GPI ANCHOR BIOSYNTHESIS	24	–0.20336	–0.65816	0.869121	0.929164
PROTEASOME	41	–0.12487	–0.47125	0.949686	0.995191

Supplementary Table S16: GSEA of KEGG-pathways in the main cohort, low Gleason (Gleason ≤ 3 + 4) samples, enrichment in ERG_{high}

PATHWAY	SIZE	ES	NES	NOM *p*-val	FDR *q*-val
NOTCH SIGNALING PATHWAY	44	0.516836	1.717935	0.001942	0.301009
AMINOACYL TRNA BIOSYNTHESIS	40	0.462563	1.56396	0.059917	0.617156
RNA DEGRADATION	52	0.354821	1.419818	0.118609	1
NITROGEN METABOLISM	16	0.588849	1.329333	0.118367	1
CITRATE CYCLE TCA CYCLE	31	0.329703	1.304396	0.142342	1
SPHINGOLIPID METABOLISM	33	0.417907	1.277418	0.118952	1
ABC TRANSPORTERS	35	0.413801	1.277238	0.148148	1
ONE CARBON POOL BY FOLATE	16	0.446341	1.241598	0.219959	1
TYPE II DIABETES MELLITUS	32	0.409184	1.20927	0.201195	1
PENTOSE PHOSPHATE PATHWAY	24	0.331508	1.207402	0.213018	1
GLYOXYLATE AND DICARBOXYLATE METABOLISM	15	0.400063	1.203525	0.245902	1
ETHER LIPID METABOLISM	23	0.443994	1.194557	0.198853	1
GLYCOSAMINOGLYCAN BIOSYNTHESIS HEPARAN SULFATE	20	0.343143	1.071101	0.349693	1
SPLICEOSOME	106	0.22487	1.047405	0.391837	1
GLYCOSAMINOGLYCAN DEGRADATION	19	0.358777	1.041911	0.423554	1
N GLYCAN BIOSYNTHESIS	43	0.283753	1.040282	0.413927	1
SMALL CELL LUNG CANCER	77	0.299533	1.031481	0.406977	1
BLADDER CANCER	41	0.317529	1.031392	0.412121	1
OLFACTORY TRANSDUCTION	131	0.321648	1.020145	0.440162	1
BASE EXCISION REPAIR	32	0.27957	1.018931	0.430672	1
PURINE METABOLISM	137	0.229031	1.012979	0.449807	1
PENTOSE AND GLUCURONATE INTERCONVERSIONS	22	0.375712	0.980591	0.516832	1
NEUROACTIVE LIGAND RECEPTOR INTERACTION	144	0.287321	0.970639	0.497154	1
DNA REPLICATION	36	0.306206	0.965192	0.480808	1
GLYCOSAMINOGLYCAN BIOSYNTHESIS CHONDROITIN SULFATE	19	0.374668	0.959322	0.517578	1
LYSINE DEGRADATION	41	0.276211	0.950357	0.538023	1
TGF BETA SIGNALING PATHWAY	71	0.323419	0.932605	0.573674	1
DORSO VENTRAL AXIS FORMATION	23	0.318803	0.913324	0.569573	1
UBIQUITIN MEDIATED PROTEOLYSIS	127	0.19352	0.909368	0.55814	1
ECM RECEPTOR INTERACTION	70	0.341929	0.900604	0.611632	1
GLYCINE SERINE AND THREONINE METABOLISM	29	0.31782	0.899822	0.619718	1
PROGESTERONE MEDIATED OOCYTE MATURATION	74	0.24859	0.88205	0.654297	1
MISMATCH REPAIR	22	0.267798	0.872425	0.608696	1
CELL CYCLE	108	0.229027	0.872099	0.646943	1
ERBB SIGNALING PATHWAY	74	0.192177	0.860207	0.731313	1
RIG I LIKE RECEPTOR SIGNALING PATHWAY	54	0.230037	0.852649	0.697495	1
OOCYTE MEIOSIS	98	0.220263	0.847816	0.732284	1
VASOPRESSIN REGULATED WATER REABSORPTION	38	0.240948	0.844498	0.646341	1
AMYOTROPHIC LATERAL SCLEROSIS ALS	44	0.281079	0.83691	0.672414	1

**INSULIN SIGNALING PATHWAY**	114	0.187648	0.79114	0.801181	1
**O GLYCAN BIOSYNTHESIS**	19	0.275072	0.784538	0.810176	1
**HOMOLOGOUS RECOMBINATION**	24	0.236923	0.777339	0.755144	1
**NEUROTROPHIN SIGNALING PATHWAY**	114	0.179373	0.77006	0.825203	1
**PROTEIN EXPORT**	22	0.236003	0.764155	0.708098	1
**CYSTEINE AND METHIONINE METABOLISM**	29	0.220384	0.753994	0.841176	1
**BASAL TRANSCRIPTION FACTORS**	28	0.19441	0.750105	0.825203	1
**SELENOAMINO ACID METABOLISM**	21	0.244671	0.747654	0.793587	0.996678
**RIBOSOME**	82	0.195386	0.737388	0.690909	0.991515
**ENDOMETRIAL CANCER**	45	0.199174	0.732926	0.799599	0.978085
**CHRONIC MYELOID LEUKEMIA**	65	0.193513	0.729681	0.813627	0.963081
**ACUTE MYELOID LEUKEMIA**	53	0.198753	0.728204	0.884086	0.946406
**TASTE TRANSDUCTION**	25	0.243484	0.68766	0.948077	0.979054
**NOD LIKE RECEPTOR SIGNALING PATHWAY**	48	0.215297	0.672106	0.900778	0.978148
**NUCLEOTIDE EXCISION REPAIR**	42	0.155527	0.611816	0.902153	1
**THYROID CANCER**	29	0.193289	0.605692	0.926878	0.993572
**REGULATION OF AUTOPHAGY**	24	0.177812	0.605173	0.937736	0.97612
**CYTOSOLIC DNA SENSING PATHWAY**	42	0.143015	0.491659	0.992481	0.99366

Supplementary Table S17: GSEA of KEGG-pathways in the main cohort, high Gleason (Gleason ≥ 4 + 3) samples, enrichment in ERG_{low}

PATHWAY	SIZE	ES	NES	NOM *p*-val	FDR *q*-val
GLUTATHIONE METABOLISM	45	–0.58109	–1.49226	0.062753	1
DRUG METABOLISM CYTOCHROME P450	55	–0.52313	–1.45852	0.018256	1
GLYCOLYSIS GLUCONEOGENESIS	56	–0.40615	–1.44262	0.053254	1
P53 SIGNALING PATHWAY	61	–0.45124	–1.38952	0.070342	1
APOPTOSIS	75	–0.33517	–1.38622	0.045455	1
BETA ALANINE METABOLISM	19	–0.53945	–1.33119	0.124	1
ARGININE AND PROLINE METABOLISM	46	–0.48317	–1.31523	0.110442	1
GLYCOSPHINGOLIPID BIOSYNTHESIS LACTO AND NEOLACTO SERIES	17	–0.46753	–1.30379	0.120623	1
HISTIDINE METABOLISM	24	–0.54079	–1.3033	0.09	1
MELANOGENESIS	82	–0.38026	–1.29089	0.093361	1
ALDOSTERONE REGULATED SODIUM REABSORPTION	33	–0.53263	–1.27956	0.136095	1
HYPERTROPHIC CARDIOMYOPATHY HCM	66	–0.43965	–1.27941	0.187373	0.9968
METABOLISM OF XENOBIOTICS BY CYTOCHROME P450	50	–0.43719	–1.25665	0.117647	1
PYRUVATE METABOLISM	38	–0.36316	–1.23956	0.208413	1
PROSTATE CANCER	83	–0.30743	–1.21731	0.155419	1
MTOR SIGNALING PATHWAY	43	–0.30711	–1.18744	0.1917	1
O GLYCAN BIOSYNTHESIS	19	–0.45238	–1.18317	0.239837	1
DILATED CARDIOMYOPATHY	73	–0.40563	–1.18086	0.264463	1
TYROSINE METABOLISM	31	–0.43806	–1.17859	0.211765	0.984351
PRION DISEASES	30	–0.44281	–1.17312	0.29505	0.957145
CARDIAC MUSCLE CONTRACTION	58	–0.34436	–1.14871	0.26938	1
PPAR SIGNALING PATHWAY	51	–0.39044	–1.12762	0.262425	1
GLIOMA	57	–0.31679	–1.12513	0.25102	1
TASTE TRANSDUCTION	25	–0.42644	–1.07992	0.373016	1
STEROID HORMONE BIOSYNTHESIS	36	–0.37043	–1.07989	0.325671	1
FATTY ACID METABOLISM	35	–0.3198	–1.07466	0.351515	1
PYRIMIDINE METABOLISM	90	–0.26562	–1.07115	0.339286	1
CYSTEINE AND METHIONINE METABOLISM	29	–0.31216	–1.0571	0.397895	1
HEMATOPOIETIC CELL LINEAGE	68	–0.42636	–1.046	0.397638	1
TRYPTOPHAN METABOLISM	33	–0.39935	–1.03939	0.393375	1
NICOTINATE AND NICOTINAMIDE METABOLISM	19	–0.4544	–1.03812	0.371717	1
VALINE LEUCINE AND ISOLEUCINE DEGRADATION	44	–0.38296	–1.03049	0.423387	1
PROXIMAL TUBULE BICARBONATE RECLAMATION	18	–0.39738	–1.0253	0.41966	0.996142
GLYCINE SERINE AND THREONINE METABOLISM	29	–0.38587	–1.01423	0.426195	1
ARACHIDONIC ACID METABOLISM	40	–0.38977	–0.99708	0.441955	1
ARRHYTHMOGENIC RIGHT VENTRICULAR CARDIOMYOPATHY ARVC	59	–0.34466	–0.98655	0.48394	1
AMYOTROPHIC LATERAL SCLEROSIS ALS	44	–0.30689	–0.96829	0.48855	1
PHENYLALANINE METABOLISM	17	–0.41873	–0.96135	0.516	1
LINOLEIC ACID METABOLISM	16	–0.39795	–0.94534	0.564777	1

NOD LIKE RECEPTOR SIGNALING PATHWAY	48	–0.31988	–0.93946	0.53229	1
OLFACTORY TRANSDUCTION	131	–0.33618	–0.93875	0.53816	1
ADHERENS JUNCTION	62	–0.25968	–0.93686	0.537473	1
FOCAL ADHESION	168	–0.29155	–0.93682	0.521739	1
TERPENOID BACKBONE BIOSYNTHESIS	15	–0.39638	–0.93531	0.560078	0.984399
PROPANOATE METABOLISM	31	–0.31655	–0.92299	0.546667	0.99571
WNT SIGNALING PATHWAY	129	–0.25453	–0.92235	0.572614	0.975564
PHOSPHATIDYLINOSITOL SIGNALING SYSTEM	65	–0.26402	–0.91989	0.566596	0.961487
RETINOL METABOLISM	40	–0.29044	–0.91814	0.634	0.945594
ALANINE ASPARTATE AND GLUTAMATE METABOLISM	28	–0.32715	–0.9138	0.59802	0.936682
CALCIUM SIGNALING PATHWAY	124	–0.2778	–0.91263	0.577963	0.9206
PATHWAYS IN CANCER	279	–0.23088	–0.9015	0.640657	0.931577
PORPHYRIN AND CHLOROPHYLL METABOLISM	33	–0.29245	–0.89562	0.598441	0.928584
AXON GUIDANCE	119	–0.25724	–0.89225	0.664	0.918983
LONG TERM POTENTIATION	60	–0.27098	–0.88623	0.655319	0.916726
CYTOSOLIC DNA SENSING PATHWAY	42	–0.28958	–0.88511	0.591356	0.902483
DRUG METABOLISM OTHER ENZYMES	39	–0.29008	–0.88207	0.672549	0.893022
PANCREATIC CANCER	63	–0.24008	–0.87586	0.667355	0.891418
DORSO VENTRAL AXIS FORMATION	23	–0.26575	–0.87206	0.633401	0.884406
VASCULAR SMOOTH MUSCLE CONTRACTION	89	–0.25807	–0.84826	0.658174	0.921096
HEDGEHOG SIGNALING PATHWAY	39	–0.30239	–0.81983	0.735537	0.968674
COLORECTAL CANCER	54	–0.22429	–0.81853	0.772257	0.955406
COMPLEMENT AND COAGULATION CASCADES	52	–0.33958	–0.8171	0.68	0.942883
BLADDER CANCER	41	–0.26128	–0.81446	0.761044	0.933214
HUNTINGTONS DISEASE	157	–0.17929	–0.81213	0.683168	0.923699
NUCLEOTIDE EXCISION REPAIR	42	–0.20196	–0.80325	0.714004	0.927309
DNA REPLICATION	36	–0.24967	–0.79526	0.678208	0.929258
CYTOKINE CYTOKINE RECEPTOR INTERACTION	183	–0.27434	–0.7898	0.749515	0.925759
CHEMOKINE SIGNALING PATHWAY	152	–0.26392	–0.77536	0.762295	0.938934
EPITHELIAL CELL SIGNALING IN HELICOBACTER PYLORI INFECTION	65	–0.1986	–0.76677	0.833652	0.941243
RENAL CELL CARCINOMA	61	–0.20876	–0.75937	0.79065	0.940721
CELL CYCLE	108	–0.19763	–0.7411	0.85567	0.957485
GALACTOSE METABOLISM	20	–0.22163	–0.73411	0.790476	0.955116
SMALL CELL LUNG CANCER	77	–0.20928	–0.72354	0.89834	0.957566
B CELL RECEPTOR SIGNALING PATHWAY	70	–0.23307	–0.72088	0.819106	0.948205
RNA POLYMERASE	27	–0.2329	–0.69867	0.792887	0.965847
CELL ADHESION MOLECULES CAMS	112	–0.25326	–0.66414	0.89	0.991479
HOMOLOGOUS RECOMBINATION	24	–0.19277	–0.6397	0.932939	1
PROTEASOME	41	–0.18083	–0.62023	0.806911	1
ANTIGEN PROCESSING AND PRESENTATION	65	–0.22812	–0.59258	0.919075	1
PARKINSONS DISEASE	103	–0.13483	–0.57441	0.925996	1
OXIDATIVE PHOSPHORYLATION	107	–0.13049	–0.55556	0.900952	0.99953
GLYCOSYLPHOSPHATIDYLINOSITOL GPI ANCHOR BIOSYNTHESIS	24	–0.17695	–0.51881	0.924419	0.998504
AMINOACYL TRNA BIOSYNTHESIS	40	–0.12514	–0.40487	0.993776	0.998799

Supplementary Table S18: GSEA of KEGG-pathways in the main cohort, high Gleason (Gleason ≥ 4 + 3) samples, enrichment in ERG_{high}

PATHWAY	SIZE	ES	NES	NOM *p*-val	FDR *q*-val
**NOTCH SIGNALING PATHWAY**	44	0.5045062	1.6738925	0	0.62938935
**PENTOSE AND GLUCURONATE INTERCONVERSIONS**	22	0.5123342	1.4166561	0.08817204	1
**GLYCOSAMINOGLYCAN BIOSYNTHESIS CHONDROITIN SULFATE**	19	0.542504	1.3943979	0.07114624	1
**SELENOAMINO ACID METABOLISM**	21	0.47533673	1.3648516	0.10714286	1
**ENDOMETRIAL CANCER**	45	0.36318752	1.3550841	0.086519115	1
**PROTEIN EXPORT**	22	0.48945096	1.3471158	0.19421488	1
**SPLICEOSOME**	106	0.31644973	1.3434168	0.17	1
**ETHER LIPID METABOLISM**	23	0.44876847	1.3325243	0.06832298	1
**GAP JUNCTION**	73	0.40344366	1.2925855	0.13872832	1
**LONG TERM DEPRESSION**	53	0.35514474	1.2659172	0.1002004	1
**TYPE II DIABETES MELLITUS**	32	0.36255944	1.2274318	0.15039062	1
**UBIQUITIN MEDIATED PROTEOLYSIS**	127	0.27910286	1.227381	0.24418604	1
**TIGHT JUNCTION**	106	0.2995418	1.1919937	0.18426104	1
**GLYOXYLATE AND DICARBOXYLATE METABOLISM**	15	0.41961658	1.1883075	0.28985506	1
**PROGESTERONE MEDIATED OOCYTE MATURATION**	74	0.329471	1.171812	0.25494072	1
**LYSINE DEGRADATION**	41	0.2979997	1.171253	0.19444445	1
**ABC TRANSPORTERS**	35	0.3798634	1.1644273	0.2576336	1
**RNA DEGRADATION**	52	0.2598646	1.1500181	0.30754352	1
**RIBOSOME**	82	0.3375702	1.129038	0.3858586	1
**GLYCEROLIPID METABOLISM**	38	0.3245482	1.1109859	0.3046092	1
**STARCH AND SUCROSE METABOLISM**	38	0.34416395	1.1031003	0.32040817	1
**GLYCEROPHOSPHOLIPID METABOLISM**	59	0.2613262	1.091921	0.28870293	1
**RIG I LIKE RECEPTOR SIGNALING PATHWAY**	54	0.3146344	1.0732448	0.35976788	1
**CITRATE CYCLE TCA CYCLE**	31	0.29832336	1.0654979	0.39300412	1
**VEGF SIGNALING PATHWAY**	61	0.3033966	1.0607156	0.37058824	1
**SPHINGOLIPID METABOLISM**	33	0.3293276	1.0508296	0.35849056	1
**PURINE METABOLISM**	137	0.26441482	1.0403163	0.39648438	1
**N GLYCAN BIOSYNTHESIS**	43	0.32496336	1.0089209	0.45759368	1
**VIBRIO CHOLERAE INFECTION**	51	0.2632098	0.99514633	0.43838385	1
**GLYCOSAMINOGLYCAN BIOSYNTHESIS HEPARAN SULFATE**	20	0.3191126	0.9949526	0.49115914	1
**GLYCOSAMINOGLYCAN DEGRADATION**	19	0.3005399	0.965958	0.5010309	1
**THYROID CANCER**	29	0.29003474	0.9657133	0.5121951	1
**BASE EXCISION REPAIR**	32	0.26781192	0.963137	0.49707603	1
**MISMATCH REPAIR**	22	0.303365	0.95994514	0.5051546	1
**ALZHEIMERS DISEASE**	141	0.23473425	0.94902	0.5030303	1
**MAPK SIGNALING PATHWAY**	218	0.2395718	0.94869226	0.560241	1
**BASAL TRANSCRIPTION FACTORS**	28	0.25926867	0.9475964	0.5235294	1
**NON SMALL CELL LUNG CANCER**	50	0.27112022	0.9459981	0.52566737	1
**VASOPRESSIN REGULATED WATER REABSORPTION**	38	0.23592153	0.93527263	0.5808967	1
**GNRH SIGNALING PATHWAY**	84	0.25247583	0.92241526	0.5753425	1
**OOCYTE MEIOSIS**	98	0.24481033	0.9178716	0.5795678	1

**ERBB SIGNALING PATHWAY**	74	0.22490917	0.9112399	0.6229508	1
**NEUROACTIVE LIGAND RECEPTOR INTERACTION**	144	0.2674146	0.90514165	0.68801653	1
**AMINO SUGAR AND NUCLEOTIDE SUGAR METABOLISM**	41	0.28475586	0.89959615	0.57758623	1
**ASCORBATE AND ALDARATE METABOLISM**	18	0.3372187	0.887614	0.6293996	1
**TGF BETA SIGNALING PATHWAY**	71	0.28805873	0.8791876	0.64176244	1
**T CELL RECEPTOR SIGNALING PATHWAY**	92	0.27073228	0.8785631	0.61044174	1
**PENTOSE PHOSPHATE PATHWAY**	24	0.27586928	0.87095606	0.6260504	1
**ENDOCYTOSIS**	156	0.20839994	0.86836064	0.6947162	1
**FC EPSILON RI SIGNALING PATHWAY**	62	0.272123	0.8679006	0.612326	1
**ADIPOCYTOKINE SIGNALING PATHWAY**	56	0.254351	0.8639951	0.70726913	1
**ECM RECEPTOR INTERACTION**	70	0.33320397	0.8552361	0.6287425	1
**TYPE I DIABETES MELLITUS**	32	0.4039086	0.84916973	0.64123714	1
**JAK STAT SIGNALING PATHWAY**	114	0.24348678	0.8405615	0.6825397	1
**SYSTEMIC LUPUS ERYTHEMATOSUS**	88	0.31594077	0.8378146	0.69896907	1
**MELANOMA**	58	0.23131153	0.8347135	0.80846775	1
**INSULIN SIGNALING PATHWAY**	114	0.19043854	0.83018816	0.74810606	1
**SNARE INTERACTIONS IN VESICULAR TRANSPORT**	34	0.24903408	0.82990885	0.7033399	1
**NITROGEN METABOLISM**	16	0.36240193	0.82630676	0.7225807	1
**PEROXISOME**	67	0.23194745	0.82561415	0.7344961	0.99797565
**REGULATION OF AUTOPHAGY**	24	0.22092693	0.82355046	0.7057654	0.9860101
**BASAL CELL CARCINOMA**	40	0.28743526	0.82145005	0.78149605	0.97474784
**CHRONIC MYELOID LEUKEMIA**	65	0.20110641	0.8113076	0.7887324	0.981718
**NEUROTROPHIN SIGNALING PATHWAY**	114	0.17418417	0.81061876	0.8224852	0.9678766
**ASTHMA**	21	0.3933154	0.79921764	0.7057654	0.9763035
**ONE CARBON POOL BY FOLATE**	16	0.32356194	0.798125	0.68604654	0.9636205
**INOSITOL PHOSPHATE METABOLISM**	49	0.2127568	0.7812778	0.8507752	0.9808415
**LYSOSOME**	117	0.21278796	0.7681107	0.8032787	0.99078923
**BIOSYNTHESIS OF UNSATURATED FATTY ACIDS**	20	0.31573716	0.72537094	0.8079332	1
**LEISHMANIA INFECTION**	58	0.28457937	0.7233578	0.8353909	1
**BUTANOATE METABOLISM**	30	0.28861216	0.7105619	0.85626286	1
**PATHOGENIC ESCHERICHIA COLI INFECTION**	50	0.24425834	0.70852464	0.8627859	1
**LEUKOCYTE TRANSENDOTHELIAL MIGRATION**	94	0.24133033	0.7067604	0.8313953	1
**REGULATION OF ACTIN CYTOSKELETON**	176	0.17993955	0.6648534	0.9596154	1
**TOLL LIKE RECEPTOR SIGNALING PATHWAY**	76	0.21081783	0.65017754	0.9047619	1
**FC GAMMA R MEDIATED PHAGOCYTOSIS**	83	0.19004686	0.6495817	0.90335304	1
**ACUTE MYELOID LEUKEMIA**	53	0.16568136	0.6410138	0.9823875	1
**FRUCTOSE AND MANNOSE METABOLISM**	30	0.19758251	0.6064993	0.95669293	1
**NATURAL KILLER CELL MEDIATED CYTOTOXICITY**	101	0.2088917	0.6036799	0.92622954	1
**PRIMARY IMMUNODEFICIENCY**	29	0.29064822	0.58000416	0.88188976	1
**ALLOGRAFT REJECTION**	26	0.3025116	0.57801336	0.93890023	1
**INTESTINAL IMMUNE NETWORK FOR IGA PRODUCTION**	34	0.261487	0.5624501	0.94949496	1
**GRAFT VERSUS HOST DISEASE**	31	0.26586834	0.5504549	0.9738956	1
**STEROID BIOSYNTHESIS**	16	0.22409485	0.55034274	0.96825397	1
**AUTOIMMUNE THYROID DISEASE**	34	0.26633847	0.5500026	0.9534413	0.98951966
**VIRAL MYOCARDITIS**	62	0.21385355	0.5458711	0.9785575	0.9793871

Supplementary Table S19: Pearson correlations between individual metabolites and selected tissue composition parameters among cancer samples in the main cohort

Metabolites	Cancer content		Stroma content		Benign epithelium content		Relative luminal space	
	Correlations	*p*-value	Correlations	*p*-value	Correlations	*p*-value	Correlations	*p*-value
**Alanine**	0.2295	0.0253	–0.2269	0.027	–0.0803	0.439	–0.0087	0.9337
**Choline**	0.5395	< 0.001	–0.5233	< 0.001	–0.3218	0.0002	–0.2185	0.0132
**Citrate**	–0.0618	0.5518	–0.1113	0.2828	0.2662	0.0091	0.436	< 0.001
**Creatine**	–0.0642	0.4699	0.0024	0.9783	0.1075	0.2254	0.0397	0.6564
**Ethm**	–0.0341	0.701	0.0277	0.7557	0.0267	0.7643	–0.0687	0.4412
**Glucose**	–0.6205	< 0.001	0.514	< 0.001	0.4716	< 0.001	0.1263	0.1554
**Glutamate**	0.5898	< 0.001	–0.4871	< 0.001	–0.45	< 0.001	–0.1672	0.0592
**Glutamine**	0.4315	< 0.001	–0.3368	0.0001	–0.3519	< 0.001	–0.2222	0.0117
**Glycine**	–0.4881	< 0.001	0.3687	< 0.001	0.4122	< 0.001	0.2114	0.0166
**GPC**	0.491	< 0.001	–0.4792	< 0.001	–0.2894	0.0009	–0.0006	0.9946
**GPE**	–0.0362	0.6839	0.0357	0.6876	0.0208	0.8146	–0.0531	0.5518
**Isoleucine**	0.2005	0.0227	–0.1439	0.1036	–0.178	0.0436	–0.1519	0.087
**Lactate**	0.4693	< 0.001	–0.4064	< 0.001	–0.338	0.0001	–0.1843	0.038
**Leucine**	0.368	< 0.001	–0.2978	0.0006	–0.2878	0.0009	–0.2375	0.007
**Myo–inositol**	0.0784	0.3771	–0.1006	0.2568	–0.0184	0.8359	–0.0644	0.4704
**PCh**	0.4436	< 0.001	–0.2875	0.001	–0.4296	< 0.001	–0.1757	0.0472
**PE**	0.3601	< 0.001	–0.2105	0.0167	–0.3752	< 0.001	–0.2391	0.0066
**Putrescine**	–0.3059	0.0004	0.0277	0.7555	0.4935	< 0.001	0.1555	0.0796
**Scyllo–inositol**	0.1097	0.216	–0.0281	0.752	–0.1559	0.0776	0.0436	0.6254
**Spermine**	–0.0485	0.6405	–0.1098	0.2897	0.2407	0.0188	0.4536	< 0.001
**Succinate**	0.374	< 0.001	–0.3517	< 0.001	–0.2358	0.0071	–0.0454	0.6106
**Taurine**	–0.0895	0.3131	0.0881	0.3211	0.052	0.5586	–0.1108	0.2129
**Valine**	0.2057	0.0193	–0.1288	0.1457	–0.2045	0.0201	–0.182	0.0398

Ethm: Ethanolamine, GPC: Glycerophosphocholine, GPE: Glycerophosphoethanolamine, PCh: Phosphocholine, PE: Phosphoethanolamine.
